# Development of novel benzamide class I selective lysine deacetylase inhibitors as potent anticancer agents

**DOI:** 10.1080/14756366.2025.2520612

**Published:** 2025-07-01

**Authors:** Jason H. Gill, Jonathan D. Sellars, Paul G. Waddell, Steven D. Shnyder, Ronald Grigg, Colin W. G. Fishwick

**Affiliations:** ^a^ School of Pharmacy, Newcastle University, Newcastle upon Tyne, UK; ^b^ Translational and Clinical Research Institute, Newcastle University, Newcastle upon Tyne, UK; ^c^ Biosciences Institute, Faculty of Medical Sciences, Newcastle University, Newcastle upon Tyne, UK; ^d^ Chemistry, School of Natural and Environmental Science, Newcastle University, Newcastle upon Tyne, UK; ^e^ Institute of Cancer Therapeutics, School of Life Sciences, University of Bradford, Bradford, UK; ^f^ School of Chemistry, University of Leeds, Leeds, UK

**Keywords:** Lysine Deacetylase (KDACs), histone deacetylase (HDACs), HDAC Inhibitor, cancer chemotherapy, aminobenzamide, protein acetylation, benzamide

## Abstract

Small molecule inhibitors of lysine deacetylases (KDACs), exemplified by histone deacetylases (HDACs), exhibit significant promise as cancer therapeutics. Using a modular combinatorial chemistry approach, a novel class of KDAC inhibitors (KDACi) containing the aminophenyl-benzamide headgroup have been developed, which incorporate a vinyl group within the linker region for active site stabilisation and a trifluoromethyl moiety within the capping group to exploit enzyme surface topology. Consequently, a class I selective KDACi (**7**) with a preference towards HDAC1 over other class I KDACs was identified. This KDACi orientates differently within the KDAC active site and exhibits an improved antitumour profile relative to the benchmark class I selective KDACi Entinostat (**1**). The clinical potential of **7** is further exemplified by the inhibition of tumour growth in an *in vivo* model of ovarian cancer. These results offer significant scope for the rational development of KDACi with improved selectivity against specific KDAC and widespread therapeutic potential.

## Introduction

Dysregulation and irregularities in the activity of lysine acetyltransferases (KATs) and lysine deacetylases (KDACs) is implicated in the development and progression of several diseases, including cancer^1–5^. KDACs remove the ε-N-acetyl group from hyperacetylated lysine residues and comprise the zinc-dependent histone deacetylases (HDACs) and the nicotinamide adenine dinucleotide (NAD^+^)-dependent sirtuin enzymes (initially termed class III HDACs)^5^. The resulting activity of KDACs is the post-translational modification of the charge on lysine residues, which thereby alters protein structure and influences target protein activity and stability^5–8^. These activities ultimately regulate a number of cellular processes including signal transduction, cell morphology, metabolism, proliferation, death and survival^1^^,^^7^^,^^9–11^. A well-established function of KDACs is the regulation of lysine residues in the tail region of nuclear histones, proteins central to the regulation of gene transcription^11^. This function of KDACs coupled to their dysregulation in diseases led to the inhibition of KDAC activity being identified as a therapeutic strategy, particularly as anticancer agents.

The zinc-dependent HDAC sub-family of KDACs is further subdivided into four classes based on their enzymatic activity and subcellular localisation^12^^,^^13^. Class I (HDACs 1, 2, 3 and 8) are localised primarily in the nucleus and are central to the regulation of gene transcription^14^^,^^15^. Class IIa (HDACs 4, 5, 7 and 9) shuttle between the cytoplasm and nucleus and have low or negligible enzymatic activity, supporting a role for them as transcriptional co-repressors^13^. Class IIb (HDACs 6 and 10) are localised primarily in the cytoplasm wherein they regulate the activity of several cytoplasmic molecules^12^^,^^16^^,^^17^. Class IV (HDAC11) share features with both class I and II but are believed to function specifically in the nucleus^12^. Based on their multifaceted roles, aberrant activation and expression of KDACs is implicated in a range of diseases and pathologies^1^^,^^18–24^. Inhibition of the activity of specific KDACs, especially HDACs, has thus received significant attention as a therapeutic strategy for the management of many diseases and clinical disorders, particularly cancers and leukaemias^13^^,^^18^^,^^24–26^. In particular, the class I sub-family KDACs (specifically HDACs 1–3), are known to play strategic and fundamental roles in cancer development and progression, gaining significant interest as clinically viable targets for KDACi development^25–31^.

The majority of KDACi developed to date exhibit a common three-component pharmacophore: a zinc-binding group (ZBG) chelating the catalytic zinc ion, a linker group which resides within the hydrophobic substrate binding tunnel, and a capping group (CAP) which interacts with the rim of the enzymatic pocket and potentially contributes to KDACi selectivity^31–36^. The archetypal KDACi incorporate hydroxamic acids as the ZBG, with three such drugs currently approved for clinical use; suberoylanilide hydroxamic acid (SAHA)/Vorinostat, Belinostat/PDX101 and Panobinostat/LBH-589, against lymphomas and myelomas^13^^,^^37–39^. Despite demonstrating clinical efficacy, a major drawback with hydroxamate-based KDACi include lack of selectivity towards specific KDAC classes and isoforms, poor pharmacokinetic properties, narrow therapeutic indices, and overt and widespread toxicities^40^. As a consequence of these limitations, agents with improved class I KDAC isoform selectivity and non-hydroxamate ZBG’s were sought^13^^,^^40^. The most promising ZBG’s in this respect are the benzamide (or 2-aminoanilides), that demonstrate selectivity for class I KDACs and significantly reduced toxicities relative to hydroxamates^13^^,^^35^^,^^40^. These KDACi are exemplified by Entinostat/MS-275 (Phase III clinical trial) (see Table 1), Mocetinostat/MGCD0103 (phase II clinical trial), Tacedinaline/CI-994 (Phase III clinical trial) and Tucidinostat/chidamide^13^^,^^41–46^. However, despite demonstrating class I KDAC specificity, differences exist between these benzamide-containing KDACi with regards to inhibitory activity against HDACs 1–3. For instance, Entinostat (**1**) demonstrates selectivity for HDAC1 over HDAC2 and HDAC3, Mocetinostat is highly active against HDAC1 and HDAC2 but less active against HDAC3, and Tacedinaline/CI-994 shows little selectivity between HDACs1–3^13,^^32^. These differential activities thus offer further scope for the development of isoform-selective KDACi as both probes to assess the contributions of a specific HDAC to disease pathophysiology and as molecular-targeted cancer therapeutics.

**Table 1.** Activity of reference class I selective KDACi.

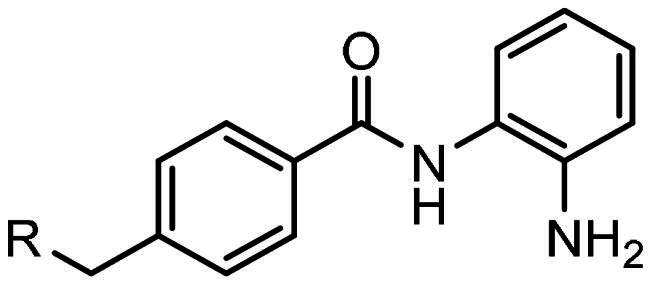						
Compound	R Group	ΔG_bind_ (kcal/mol)	pLogD_7.4_	HDAC Inhibition IC_50_ (µM)	A2780 IC_50_ (µM)	MCF7 IC_50_ (µM)
1 Entinostat (MS-275)	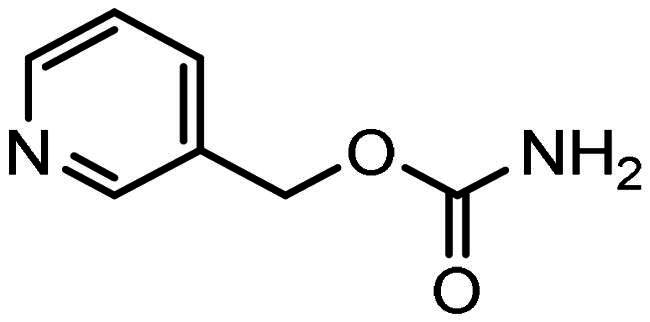	−11.6	1.92	3.0 ± 0.2	0.6 ± 0.04	0.4 ± 0.1
2 Tacedinaline (CI-994)	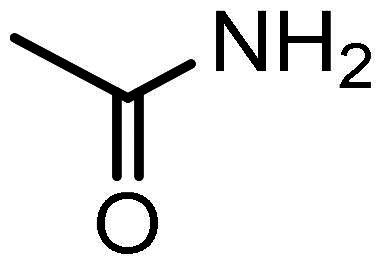	−11.1	1.17	*8.5 ± 5.4* ^a^	12.0 ± 2.5	11.6 ± 1.1

Inhibition of HDAC activity (as determined by Fluor-de-Lys assay), calculated binding energy (ΔG_bind_) by Autodock 3.0 of compounds against HDLP, pLogD_7.4_ values obtained from ChemSpider (http://www.chemspider.com), generated using the ACD/Labs Percepta platform-Physchem module, and antiproliferative activity against human A2780 ovarian and MCF7 breast cancer cell lines.

^a^Data for HDAC inhibition of **2** Obtained from Beckers et al.^46^.

The class I KDAC enzymatic pocket is referenced as being analogous to the shape of a sock, with the Zn(II) ion located at the “heel”, the acetate release channel representing the “foot and toe” running perpendicular to the active site tunnel “leg”, and the active-site access located at the “exit” of the sock^31^^,^^35^. The “foot and toe” pocket is unique to class I KDACs, with the benzamide headgroup of class I selective KDACi occupying the “foot” pocket and chelating the Zn(II) through bidentate coordination of their amino group and the amide-carbonyl oxygen^13^^,^^35^^,^^47^^,^^48^. The hydrophobic active site tunnel (“leg”) is generally well conserved across KDAC isoforms with the KDACi linker residing in this area^35^. Differences between KDAC isoforms exist at the surface rim of the opening of the enzymatic cavity (“exit”), with the exploitation of topological variances in this area often proposed for the differential selectivities of KDACi^31^^,^^35^. In the majority of class I selective KDACi identified and modelled to date, the capping group reportedly exhibits a defined common orientation located within a “groove” on the external surface of the enzyme^35^. However, differences do exist between the class I KDACs in this area^31^. These subtle differences thereby offer an opportunity for the development of KDAC class I isotype selective KDACi^31^.

We herein report on the rational design, synthesis, chemical characterisation and biological evaluation of a new series of benzamide-containing KDACi’s with selectivity between HDACs 1–3, through a focus on improving interactions within the hydrophobic substrate binding (“leg”) tunnel and rim of the HDAC enzymatic pocket. With the aim of improving interactions in the hydrophobic leg tunnel and introducing a degree of stability within this region, compounds were generated incorporating an unsubstituted vinyl moiety adjacent to the aromatic ring within their linker region. Exploitation of the topological surface of the enzyme active site towards KDAC-subtype selectivity was attempted by elongating the compound through the inclusion of a piperazine moiety coupled to the terminal capping group comprising either a trifluoromethyl substituted aromatic or heteroaromatic pyridine ring.

## Materials and methods

### General chemical synthetic procedure

**Figure 1.**
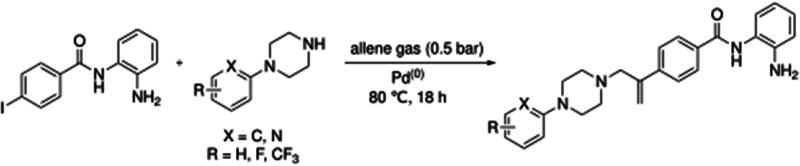
General scheme for compound synthesis.

All compounds were synthesised by methods involving catalytic cascade chemistry (Figure 1)^49^^,^^50^. The general procedure for synthesis is as follows: Nucleophile (1–3 mol eq.), tris(dibenzylideneacetone)dipalladium(0) (2.5 mol%), tris(2-furyl)phosphine (10 mol%) and base (2–4 mol eq.) were added to a solution of an aryl halide (1 mol eq.) in dry solvent (5–10 ml) in a Schlenk tube. The reaction mixture was degassed using the freeze, pump, thaw technique, before allene gas (0.5 bar) was introduced. The reaction mixture was heated at the desired temperature. After cooling, allene was vented, inorganic materials were removed by filtration, the filtrate was concentrated *in vacuo*, and the residue purified.

Chemical analyses were conducted as per our previous studies^51^, with flash column chromatography performed using Merck silica gel 60 (230–400 mesh), melting points determined using a Reichert hot-stage apparatus (remaining uncorrected), infra-red spectra recorded on a Nicolet Magna FT-IR 560 spectrometer, and microanalyses obtained using a Carbo Erba MOD 11016 Elemental Analyser. NMR spectra were obtained on a Bruker AC-250, DPX-300, or DRX-500 operating at 250, 300 or 500 MHz. Unless otherwise stated, the solvent for NMR was deuterochloroform with tetramethylsilane (TMS) as an internal standard. Chemical shifts are reported as parts per million (d) downfield from TMS, with the coupling constants determined in Hertz (Hz). Mass spectrometry were performed at Leeds University or by the EPSRC Swansea Mass Spectroscopy Service, with the mode of ionisation being EI unless otherwise stated, with EI and FAB obtained using a VG-Autospec operating at 70 eV. ES were obtained on a LCT Micromass (TOF), with accurate molecular weights determined using perfluorotributylamine or polyethylenimine as an internal standard. For all analyses, commercially available reagents and solvents were used without further purification.

#### Synthesis of (3): N-(2-aminophenyl)-4–(3-(4–(2-(trifluoromethyl)phenyl)piperazin-1-yl)prop-1-en-2-yl)benzamide


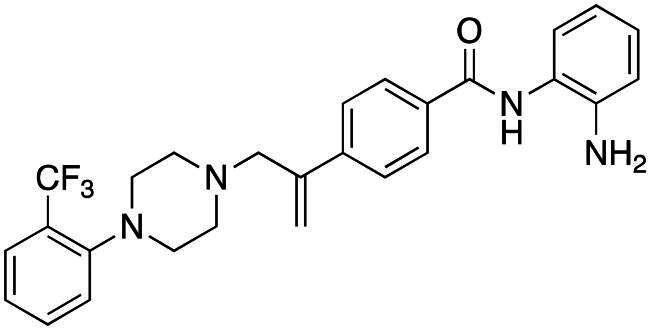


Prepared by the general procedure using N-(2-aminophenyl)-4-iodobenzamide (170 mg, 0.5 mmol), 2-[(trifluromethyl)phenyl]piperazine (126 mg, 0.55 mmol), allene (0.5 bar), tris(dibenzylideneacetone)dipalladium(0) (13 mg, 2.5 mol%), tri-2-furylphosphine (13 mg, 10 mol%), potassium carbonate (138 mg, 1 mmol) in acetonitrile (5 ml) at 80 °C for 18 h. Column chromatography of the crude product eluting with 1:1 v/v ethyl acetate/hexane afforded the title compound as colourless needles (129 mg, 54%), m.p. 167 °C–168 °C; *ν*_max_ 3340, 3242, 2943, 1622, 1603, 1557, 1524, 1495, 1455 cm^−1^; δ_H_ (500 MHz, CDCl_3_) 8.42 (1H, bs, N*H*), 7.91 (2H, d, *J* 8, Ar-*H*), 7.70 (2H, d, *J* 8, Ar-*H*), 7.64 (1H, m, Ar-*H*), 7.57 (1H, m, Ar-*H*), 7.44 (1H, m, Ar-*H*), 7.28 (1H, m, Ar-*H*), 7.21 (1H, m, Ar-*H*), 7.06 (1H, m, Ar-*H*), 6.81 (1H, m, Ar-*H*), 6.72 (1H, m, Ar-*H*), 5.62 (1H, m, C = C*H*H), 5.38 (1H, m, C = CH*H*), 4.25 (2H, bs N*H*_2_), 3.46 (2H, s, C*H*_2_C = C), 2.82 (2H, bs, NC*H*_2_), 2.35 (2H, bs, NC*H*_2_); δ_C_ (125 MHz, CDCl_3_) 165.5, 151.8, 144.1, 143.5, 141.1, 133.4, 131.8 (q, C-F), 130.0, 127.9, 127.7, 127.1, 125.6, 125.0, 120.2, 119.1, 118.8, 117.9, 116.1, 112.6, 112.5, 63.3, 53.2, 49.1; *m/z* (ES) 481 (MH^+^); Elemental Analysis: Found C, 67.5%; H, 5.65%; N, 11.7%; F, 11.9%; C_27_H_27_N_4_OF_3_ requires; C, 67.4%; H, 5.65%; N, 11.7%; F, 11.9%.

#### Synthesis of (4): N-(2-aminophenyl)-4–(3-(4–(3-(trifluoromethyl)phenyl)piperazin-1-yl)prop-1-en-2-yl)benzamide


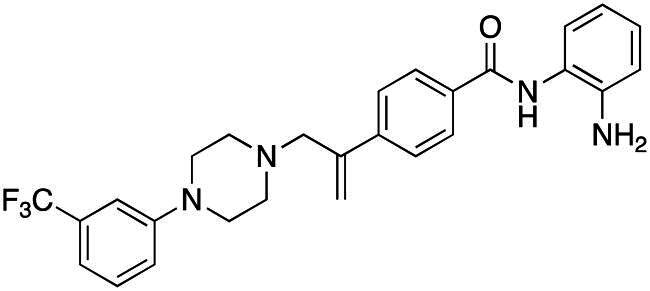


Prepared by the general procedure using N-(2- amino-phenyl)-4-iodo-benzamide, (200 mg, 0.59 mmol), 1-(trifluoro-m-tolyl)piperazine (0.122 ml, 1.1 mol eq.), potassium carbonate (163 mg, 2.0 mol eq.), tri-2-furylphosphine (14 mg, 10 mol%), tris(dibenzylideneacetone)dipalladium(0) (14 mg, 2.5 mol%) and allene gas (1 atm, 25 °C) in acetonitrile (10 ml). The Schlenk tube was heated at 80 °C for 22.5 h to give the title compound that was purified by gradient flash chromatography, eluting with ether (800 ml) and thereafter 19:1 (v/v) ether-methanol (R_f_ 0.05) as colourless plates (236 mg, 83%), m.p. 94–96 °C; *ν*_max_ 3423 (CONH), 1631 (CONH), 1607 (C = C) cm^−1^; δ_H_ (500 MHz, CDCl_3_) 7.88 (2H, d, *J* 8, Ar-*H*), 7.68 (2H, d, *J* 8, Ar-*H*), 7.35–7.31 (2H, m, Ar-*H*), 7.10–7.04 (4H, m, Ar-*H*), 6.87–6.80 (2H, m, Ar-*H*), 5.62 (1H, s, C = C*H*H), 5.39 (1H, s, C = CH*H*), 3.87 (2H, bs, N*H*_2_), 3.44 (2H, s, C*H*_2_C = C), 3.21 (4H, t, *J* 5, NC*H*_2_), 2.65 (4H, t, *J* 5, NC*H*_2_); δ_C_ (125 MHz, CDCl_3_) 165.5, 151.8, 144.1, 143.5, 141.1, 133.4, 131.8, 129.9, 127.9, 127.7, 127.1, 125.6, 125.0, 120.2, 119.1, 118.8, 117.9, 116.1, 112.5, 63.3, 53.2, 49.1; m/z (ES) 481 (MH^+^); Elemental Analysis: Found: C, 67.5%; H, 5.66%; N, 11.7%; F, 11.9%. C_27_H_27_N_4_ requires: C, 67.4%; H, 5.65%; N, 11.7%; F, 11.9%.

#### Synthesis of (5): N-(2-aminophenyl)-4–(3-(4–(4-(trifluoromethyl)phenyl)piperazin-1-yl)prop-1-en-2-yl)benzamide


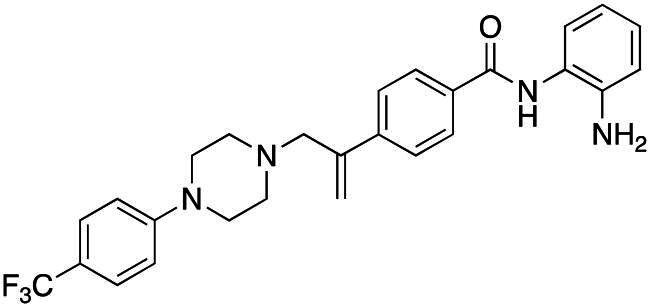


Prepared by the general procedure using N-(2-aminophenyl)-4- iodobenzamide (170 mg, 0.5 mmol), 4-[(trifluromethyl)phenyl]piperazine (126 mg, 0.55 mmol), allene (0.5 bar), tris(dibenzylideneacetone)dipalladium(0) (13 mg, 2.5 mol%), tri-2-furylphosphine (12 mg, 10 mol%), potassium carbonate (138 mg, 1 mmol) in acetonitrile (5 ml) at 80°C for 18 h. Column chromatography of the crude product eluting with 1:1 v/v ethyl acetate/hexane afforded the title compound as colourless plates (156 mg, 65%), m.p. 169–171°C; *ν*_max_ 3343, 3250, 2956, 1612, 1505, 1455 and 1390 cm^−1^; δ_H_ (500 MHz, CDCl_3_) 7.89 (2H, d, *J* 8, Ar-*H*), 7.86 (1H, bs, N*H*), 7.80 (2H, d, *J* 8, Ar-*H*), 7.46 (2H, d, *J* 9, Ar-*H*), 7.35 (2H, d, *J* 9, Ar-*H*), 7.12 (1H, m, Ar-*H*), 6.90 (3H, m, Ar-*H*), 5.62 (1H, bs, C = CH*H*), 5.38 (1H, bs, C = C*H*H), 3.90 (2H, bs, N*H*_2_), 3.42 (2H, s, C*H*_2_C = C), 3.24 (4H, t, *J* 5, NC*H*_2_), 2.65 (4H, t, *J* 5, NC*H*_2_); δ_C_ (125 MHz, CDCl_3_) 166.0, 155.7, 144.1, 143.6, 141.2, 133.4, 131.8 (q, C-F), 130.0, 127.9, 127.7, 127.1, 125.6, 125.0, 120.2, 119.1, 118.8, 117.9, 117.2, 63.4, 53.3, 50.9; *m/z* (ES) 481 (MH^+^); Elemental Analysis: Found; C, 67.6%; H, 5.55%; N, 11.7%; F, 12.0%; C_27_H_27_N_4_OF_3_ requires; C, 67.4%; H, 5.65%; N, 11.7%; F, 11.9%.

#### Synthesis of (6): N-(2-aminophenyl)-4–(3-(4-(pyridin-2-yl)piperazin-1-yl)prop-1-en-2-yl)benzamide


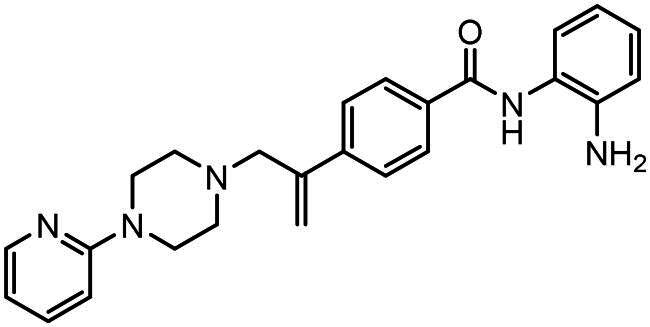


Prepared by the general procedure using N-(2- amino-phenyl)-4-iodo-benzamide (170 mg, 0.5 mmol), 1–(2-pyridyl)piperazine (0.084 ml, 1.1 mol eq.), potassium carbonate (140 mg, 2.0 mol eq.), tri-2-furylphosphine (12 mg, 10 mol%), tris(dibenzylideneacetone)dipalladium(0) (12 mg, 2.5 mol%) and allene gas (1 atm, 25°C) in acetonitrile (10 ml). The Schlenk tube was heated at 80°C. for 22 h to give the title compound that was purified by flash chromatography, eluting with 19:1 (v/v) ether-methanol (R_f_ 0.01) and thereafter by crystallisation from dichloromethane/hexane to give colourless prisms (161 mg, 78%), m.p 111–112°C, *ν*_max_ 3302, 1649, 1621 cm^−1^; δ_H_ (300 MHz, CDCl_3_) 8.18 (1H, dd, *J* 5, Ar-*H*), 8.09 (1H, s, Ar-*H*), 7.84 (2H, d, *J* 8, Ar-*H*), 7.63 (2H, d, *J* 8, Ar-*H*), 7.48–7.38 (1H, m, Ar-*H*), 7.27 (1H, d, *J* 9, Ar-*H*), 7.06 (1H, d, *J* 8, Ar-*H*), 6.82 − 6.78 (1H, m, Ar-*H*), 6.63 − 6.53 (2H, m, Ar-*H*), 5.60 (1H, s, C = CH*H*), 5.36 (lH, s, C = C*H*H), 3.49 (4H, t, *J* 5, NC*H*_2_), 3.40 (2H, s, C*H*_2_C = C), 2.57 (4H, t, NC*H*_2_); δ_C_ (75 MHz, CDCl_3_) 160.0, 148.3, 144.2, 143.5, 141.2, 137.9, 133.4, 127.7, 127.6, 127.1, 125.6, 125.0, 123.9, 120.2, 118.8, 117.8, 113.7, 107.5, 63.4, 53.2, 45.6; *m/z* (ES^+^) 414 (MH^+^); Elemental Analysis: Found C, 72.4%; H, 6.55%; N, 16.6%. C_25_H_27_N_5_ requires: C, 72.6%; H, 6.58%; N, 16.9%.

#### Synthesis of (7): N-(2-aminophenyl)-4–(3-(4–(6-(trifluoromethyl)pyridin-2-yl)piperazin-1-yl)prop-1-en-2-yl)benzamide


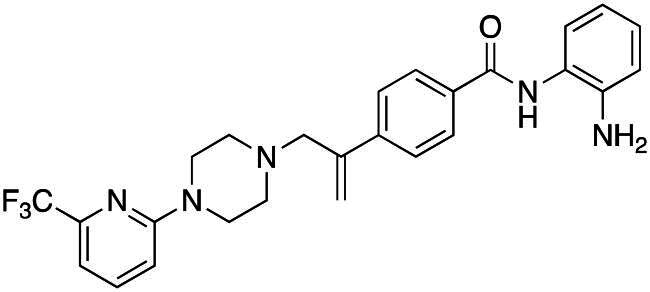


Prepared by the general procedure using N-(2-aminophenyl)-4- iodobenzamide (170 mg, 0.5 mmol), 1–(6-(trifluoromethyl)pyridin-2-yl)piperazine (126 mg, 0.55 mmol), allene (0.5 bar), tris(dibenzylideneacetone)dipalladium(0) (13 mg, 2.5 mol%), tri-2-furylphosphine (12 mg, 10 mol%), potassium carbonate (138 mg, 1 mmol) in acetonitrile (5 ml) at 80°C for 18 h. Column chromatography of the crude product eluting with 1:1 v/v ethyl acetate/hexane afforded the title compound as a beige solid (151 mg, 63%); *ν*_max_ 3322 (CONH), 1702 (CONH), 1610 (C = C) cm^−1^; δ_H_ (300 MHz, d_6_-DMSO) 9.65 (1H, bs, N*H*), 7.98 (2H, d, *J* 8, Ar-*H*), 7.76–7.67 (3H, m, Ar-*H*), 7.17 (1H, d, *J* 8, Ar-*H*), 7.10 (1H, d, *J* 8, Ar-*H*), 7.05–6.93 (2H, m, Ar-*H*), 6.78 (1H, dd, *J* 8, 1, Ar-*H*), 6.61 (1H, t, *J* 8, 1, Ar-*H*), 5.66 (1H, s, C = C*H*H), 5.38 (1H, s, C = CH*H*), 4.90 (2H, bs, N*H*_2_), 3.53 (4H, t, *J* 5, NC*H*_2_), 3.44 (2H, s, C*H*_2_C = C), 2.51 (4H, t, *J* 5, NC*H*_2_); δ_C_ (125 MHz, d_6_-DMSO) 165.3, 151.2, 143.9, 143.5, 141.1, 133.4, 131.8, 129.3, 127.9, 127.3, 126.8, 125.6, 125.0, 120.2, 119.1, 118.8, 117.9, 116.1, 112.5, 62.7, 52.7, 44.8; *m/z* (ES^+^) 482 (MH^+^). (NMR Spectra is provided as Supplementary Figure S1); Elemental Analysis Found C, 64.79%; H, 5.34%; N, 14.44%. C_26_H_26_N_5_ requires: C, 64.9%; H, 5.44%; N, 14.54%.

### Single crystal X-ray crystallography

Crystals of **4** suitable for single crystal diffraction analysis were grown via slow evaporation of the solvent from a solution of the compound in acetonitrile. Data for **4** were collected on an Xcalibur, Atlas, Gemini ultra diffractometer using copper radiation (λCuKα = 1.54184 Å). Data were collected at 150 K using an Oxford Cryosystems CryostreamPlus open-flow N2 cooling device. Intensities for K-L were corrected for absorption using a multifaceted crystal model created by indexing the faces of the crystal for which data were collected^52^. Cell refinement, data collection and data reduction were undertaken via the software CrysAlisPro^53^. All structures were solved using XT and refined by XL using the Olex2 interface^54–56^. All non-hydrogen atoms were refined anisotropically and hydrogen atoms were positioned with idealised geometry, with the exception of those bound to heteroatoms, the positions of which were located using peaks in the Fourier difference map. The displacement parameters of the hydrogen atoms were constrained using a riding model with U(H) set to be an appropriate multiple of the Ueq value of the parent atom.

### In silico chemical studies

The drug-likeness and the physiochemical properties of compounds ***1–7*** were determined using the Molinspiration web server (https://www.molinspiration.com), incorporating consideration of Lipinski’s rule of five (RO5)^57^ and Veber’s rules, respectively (Supplementary Table S1). The standard SMILES profiles of each compound were uploaded for the analyses. This analysis considered compound molecular weight (MW), the number of hydrogen-bond donors and acceptors (nHBD, nHBA), the logarithm of partition coefficient tested between *n*-octanol and water (LogP), the number of violations of RO5 (N_vio_)^57^, the topological polar surface area (TPSA), and a number of rotatable bonds (nRotB). Furthermore, to more accurately consider lipophilicity and take account of both ionised and non-ionised forms of the compounds, their predicted logarithm distribution coefficient tested between *n*-octanol and buffer at physiological pH 7.4 (pLogD_7.4_) was calculated using ChemSpider (https://www.chemspider.com).

Pharmacological absorption-distribution-metabolism-excretion (ADME) parameter profiles of compounds **1–7** were predicted using a package of online software packages; SwissADME, preADMET and psKCM^58–60^ (Supplementary Table S2). Prediction of absorption was determined via aqueous solubility (log S), both in water and buffered at pH7.4, and human intestinal absorption (HIA, %) parameters. The potential as substrates of cytochrome p450 (CYP) enzymes CYP2D6 and CYP3A4 and/or inhibitors of CYP2D6, CYP3A4, CYP1A2, CYP2C19 and CYP2C9 were determined as indicators of drug metabolism. Total body clearance (CL_tot_) was predicted as an indicator of drug excretion. An indication of the potential for induction of adverse cardiac effects was determined via the prediction of inhibition of the hERG cardiac channel.

### Molecular modelling of compounds against HDAC 1 and HDAC2

The docking of compounds **1**, **3–5** and **7** was performed using the SeeSAR molecular docking programme^61^. The protein crystal structures of HDAC 1 in complex with the dimeric ELM2-SANT domain of MTA1 (PDB: 4BKX, apo-form) and HDAC2 in complex with a 2-substituted benzamide inhibitor (PDB:7KBH, 2.68 Å) were utilised in this docking study. For clarity during docking, the domain of MTA1 was removed from HDAC1, whilst for HDAC2 the focus was on the A chain, with chains B and C along with their associated ligands removed. In all cases, water and other metals present outside of the region of interest were also excluded.

The binding sites in HDAC1 and HDAC2 were identified using the binding site module in SeeSAR. For HDAC2, SeeSAR utilised the ligand for reference to determine the active site to contain 37 residues, including the Zn(II) metal ion with 23 donor and 24 acceptor residues, a surface area of 419 Å^2^ and a volume of 401 Å^3^. The protein surface of the identified binding site revealed the characteristic “foot” and “leg” motifs with good coverage of the external surface region of the protein. For HDAC1, where a ligand available to identify the binding region was absent, the SeeSARs binding site module was used to identify the most relevant site. This identified a Zn(II) containing pocket with a familiar shape (“foot-heel-toe”) generated from 34 amino acid residues and a similar surface area of 382.7 Å^2^ and a volume of 342 Å^3^ to HDAC2. Manual addition of several surface amino acids (beyond the applied 6 Å cut-off) was performed to produce a suitable binding site for docking.

Compounds **1**, **3–7** we entered into the SeeSAR docking module and non-covalently bound poses were generated, with parameters set to produce 100 poses for each molecule, a medium clash tolerance and flexibility in aliphatic ring structures.

### Cell culture

The human A2780 ovarian (#93112519) and MCF-7 breast (#86012803) cancer cell lines were obtained from the European Cell Culture Collection (ECACC; Salisbury, UK). The human cardiac ventricular AC16 cell line (#SCC109) was obtained from Merck/Millipore (UK). The A2780 and MCF-7 cell lines were maintained in RPMI-1640 medium and the AC16 cell line was maintained in DMEM/F12 medium. Culture medium of all three cell lines was supplemented with 10% heat-inactivated foetal bovine serum (FBS) and 2 mM L-glutamine, with the cells maintained in a humidified 5% CO_2_ atmosphere at 37 °C. The media was changed every 72h and the cells passaged via trypsinisation (Trypsin EDTA, 0.25%) before reaching confluency.

### Cellular viability assay

Cytotoxicity of each compound against the cell lines was assessed using the MTT assay^62^. Cells were seeded in three replicates into 96-well plates at a density of 2 × 10^3^/well for 24h to facilitate cell attachment. Media was replaced with that containing either test compound (0–10µM) for A2780 and MCF7 cell lines, single concentration (10 µM) test compound for AC16 cell line, or drug vehicle (dimethyl sulphoxide; DMSO) and the cells incubated for a further 96 h at 37 °C in a humidified 5% CO_2_ atmosphere. In all cases, cell exposures to DMSO did not exceed 0.1%, which was confirmed to be non-cytotoxic. Chemosensitivity was thereafter assessed using a standard 3-[4, 5-dimethylthiazolyl]-2,5-diphenyltetrazolium bromide (MTT) assay^62^, with the optical density determined at 540 nm as an indicator of cellular viability. Using dose response curves, growth inhibition 50 (GI_50_; concentration required to inhibit cell growth by 50%, relative to control) values were determined, representing data from at least three independent experiments.

### NCI-60 human tumour cell line cytotoxicity screen

Compounds **3–7** were submitted to the US National Cancer Institute (NIH, Bethesda, Maryland) for screening against the NCI-60 panel of human cancer cell lines, as per standard protocol^63^. Cells were treated with compounds for 48 h across a 5-concentration range of 0.01–100 µM from which GI_50_ was determined^63^. Heat maps were constructed based on GI_50_ values; red, amber, green, blue, and white being indicative of <1µM, 1–5 µM, 5–10 µM, 10–100 µM and >100 µM, respectively.

COMPARE analyses were conducted to quantitatively assess the mean response pattern of tested compounds against those in the NCI/DTP standard agent database and synthetic agents database^64^. Matrix COMPARE coefficients (*ρ* values) of >0.7 were applied to indicate stronger potential for mechanistic correlation. The NSC numbers used to retrieve the data for this analysis were: **1**(entinostat), NSC756642; compound **3**, NSC740121; compound **4**, NSC730003; compound **5**, NSC740122; compound **6**, NSC729999; compound **7**, NSC742611.

### In vitro KDAC enzyme inhibition assay

Total zinc-dependent KDAC (classes I, II and IV) activity was measured using the Fluor-de-Lys^TM^ HDAC fluorometric activity assay kit (BioMol, Exeter, UK), as per manufacturers protocol. Briefly, HeLa nuclear extract, potential inhibitor (5 nM–20 μM) and assay buffer were incubated in a 96-well fluorometric plate at 37 °C for 15 min. Acetylated substrate (100 μM) was added and the mixture incubated at 37 °C for a further 20 min. The developer was added and the mixture incubated at 37 °C for a further 15 min. Fluorescence was measured using a microplate reader (λ_ex_ = 360 nm, λ_em_ = 460 nm). Percentage KDAC activity and thus KDAC inhibition was determined by quantification of fluorescence obtained in the presence versus absence of the respective inhibitor.

### Inhibition of activity of individual KDACs

Inhibitory profiles of compound **7** against individual zinc-dependent KDACs were performed by Reaction Biology Corporation (Malvern, PA)^65^. Test compounds (0–20 μM) or the reference compounds Trichostatin A (non-selective KDACi; 0–10 μM) and TMP269 (class II selective KDACi, 0–10 μM) were incubated in triplicate with recombinant HDACs 1–11 in assay buffer (50 mM Tris-HCl, pH 8.0, 137 mM NaCl, 2.7 mM KCl, 1 mM MgCl_2_, 1 mg/mL BSA), with the class-specific fluorogenic peptide added to initiate the reaction. After incubation for 1–2 h, the reaction was terminated by the addition of a developer solution containing trypsin. KDAC activity was determined by monitoring of the resulting fluorescence at 460 nm (λ_ex_ 360 nm)^66^. Data was graphed and IC_50_ values calculated using Prism 4 based on a sigmoidal dose-response equation (GraphPad Software, San Diego, California USA).

### Western blot analysis of cellular protein acetylation status

A2780 cells were treated with the respective compound (0–10 µM) for 24 h. Cells were then washed with ice-cold phosphate buffered saline (PBS) and lysed and sonicated in RIPA buffer [150 mM NaCl, 50 mM TrisHCl pH 7.4, 5 mM EDTA, 1% NP40, 0.1% SDS, 1 mM phenylmethylsulphonyl fluoride (PMSF) and proteinase inhibitor cocktail (1x)]. Cellular debris was removed by centrifugation (12,000 × g for 20 min, 4 °C) and protein concentration of the supernatants determined by Bradford Assay. Proteins (30 μg protein per lane) were resolved by SDS-PAGE, then transferred to the PVDF membrane (Hybond-P; Amersham, UK). For band size determination, Amersham Full-Range Rainbow^™^ Molecular Weight Markers (#RPN800) were loaded adjacent to the proteins within the gel. Non-specific protein binding on the PVDF membrane was blocked using 5% non-fat milk powder and the membranes probed separately with either anti-hyperacetylated Histone H4 (Penta) antibody (Upstate Biotechnology, USA), acetylated tubulin or β-actin antibodies (Sigma-Aldrich, UK). This was followed by incubation with horseradish peroxidase (HRP)-conjugated secondary antibodies (Dako, USA), with immunoreactive bands detected by enhanced chemiluminescence (ECL Plus detection kit; Amersham, UK).

### Flow cytometric analysis of intracellular histone acetylation status

The method was adapted from Ronzoni *et al.*^67^ as previously described^68^. A2780 cells were exposed to test compounds (0–10 µM) for 24 h, fixed with ice-cold ethanol (70% v/v) and permeabilised in the presence of 0.1% Triton-X. Non-specific protein binding was blocked using normal goat serum prior to incubation with or without primary antibodies raised against either anti-hyperacetylated Histone H4 (Penta) antibody (Upstate Biotechnology, USA) or acetylated tubulin (Sigma-Aldrich, UK), followed by incubation with FITC-conjugated secondary antibody (Dako). Cells were incubated in propidium iodide (PI) staining buffer (PBS with 50 µg/ml PI and 10 µg/ml RNase A) and subsequently analysed by flow cytometry (BD FACSCalibur; Becton Dickinson, UK). At least 15 000 events per sample were acquired and expression determined based on the detection of FITC fluorescence through the FL1 channel (530/30 nm bandpass optical filter), with cell doublets discriminated by forward/side scatter and background fluorescence omitted via comparison to unlabelled controls. Expression analysis was performed using CellQuest^™^ software (Becton Dickinson, UK).

### In vivo evaluation of anticancer efficacy

The progression of a putative cancer therapeutic from *in vitro* studies to evaluation in human clinical trials requires prior assessment of potential adverse drug-related events, safe dosing limits, and the potential for systemic efficacy *in vivo* in animal models^69^. Therefore, human tumour xenograft studies in immunodeficient nude mice were performed to evaluate compound **7**.

Female CD-1 (Crl:CD1-*Foxn1^nu^)* immunodeficient nude mice aged 6–12 weeks were used (Charles River, Margate UK). Mice were kept in stainless steel cages housed in isolation cabinets in an air-conditioned room with regular alternating cycles of light and darkness, receiving Teklad 2018 diet (Envigo, Blackthorn, UK) and water *ad libitum.* Before experimentation, all mice were allowed to acclimatise for one week to their environment. Environmental enrichment was provided in the form of Perspex “mouse houses”, chewing blocks and exercise wheels.

This study was conducted under a United Kingdom Home Office authorised Project Licence (PPL 40/2585) following local approval from the University of Bradford Animal Welfare and Ethical Review Board, with UK National Cancer Research Institute Guidelines for the Welfare of Animals (UKCCCR) and ARRIVE guidelines followed throughout^69^^,^^70^.

For evaluation of the maximum tolerated dose (MTD), two mice were treated at each of the three dose levels evaluated (total 12 mice). Compound **7** was dissolved in 15% DMSO/arachis oil and administered intraperitoneally (i.p.) as a single dose on five consecutive days with the first day designated day 0. Mice were observed daily for signs of deleterious effects and bodyweight measured frequently. A > 15% body weight loss over a 72-h period or significant changes in behaviour or appearance resulted in mouse termination. If no deleterious effects were seen after at least 14 days of study, then the animals were euthanised by isoflurane anaesthesia followed by cervical dislocation, and the dose considered non-toxic.

To assess *in vivo* compound efficacy, mice were implanted subcutaneously in the abdominal flanks with 2–3 mm^3^ ­fragments of A2780 human ovarian tumour xenograft tumours, whilst under brief general inhalation anaesthesia (2% isoflurane). Treatments were initiated when tumours reached approximately 32 mm^3^ and mice randomly assigned to cohorts (Twelve mice; *n =* 6 per group). Compound **7** (100 mg/kg) or drug vehicle (10% DMSO/arachis oil) were administered once daily via intraperitoneal (i.p.) injection on days 0–4 and 7–11. Tumour volume, via calliper measurements, and body weight were assessed daily from initiation of treatment. The cut-off points for in-study termination being a > 15% body weight loss over a 72-h period and the tumour not exceeding 15 mm in the longest diameter. All mice were euthanised by isoflurane anaesthesia followed by cervical dislocation

Calliper measurements of tumours were converted to mean tumour volume, normalised to the respective volume on day 0, and semi-log plots of relative tumour volume (RTV) versus time made. Mann–Whitney *U* tests were performed to determine the statistical significance of any differences in growth rate (based on tumour volume doubling time, RTV2) between control and treated groups.

## Results and discussion

### De novo design and synthesis of KDAC inhibitors

Compounds, based on the Entinostat (**1**) pharmacophore, were designed incorporating the 2-acylaminoaniline headgroup, but with an unsubstituted vinyl group within the linker region, and a trifluoromethyl-substituted aromatic (**3–5**) or heteroaromatic capping group (**6–7**). The potential of such compounds to act as class I selective KDACi was aided and addressed by *in silico de novo* molecular design, specifically SPROUT, an approach we have used previously for the development of enzyme inhibitors^71–74^. This software package firstly generates a molecular “skeleton” which satisfies the steric constraints of the ligand binding pocket, which in the case was defined from the crystal structure of the HDAC homologue, histone deacetylase-like protein (HDLP) from *Aquifex aeolicus* complexed with the pan-HDAC inhibitor Trichostatin A (PDB code 1C3P; 1.8 Å)^75^. Secondly, generation of the putative molecule(s) is achieved by atom-substitution to the skeleton, linking these together by spacer fragments^71^. The predicted affinity of putative KDACi were then calculated as binding “scores” using the HIPPO module within SPROUT^71^. Further information on the different modules within SPROUT can be found in Supplementary Table S3.

**Figure 2.**
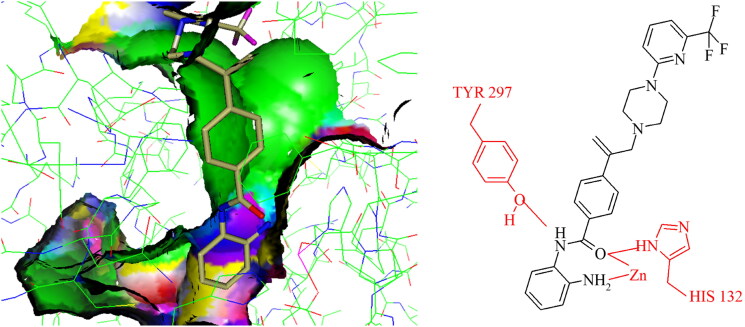
Positioning of compound 7 into the binding site of HDAC homologue, histone deacetylase-like protein (HDLP). The crystal structure of HDLP from Aquifex aeolicus complexed with the pan-HDAC inhibitor Trichostatin A (PDB code 1C3P; 1.8Å) was used for *de novo* design of KDACi using SPROUT. This configured the binding mode as “heel-leg-exit”, analogous to that for benzamide-headgroup KDACi.

All compounds **3–7** were predicted to exhibit good occupancy within the active site, with binding to the zinc atom via coordination of the carbonyl- and amino groups of the benzamide headgroup (Figure 2). In terms of orientation, this *in-silico* generated model demonstrated the binding mode of these molecules to be “heel-leg-exit” as opposed to a “toe-foot-heel-leg” conformation, with the zinc ion located at the “heel”. This binding mode is analogous to that demonstrated for Entinostat (**1**) and other benzamide-headgroup KDACi^13^^,^^35^^,^^47^^,^^48^. The introduction of the vinyl group within the linker regions of **3–7** did not retard the docking of these compounds within the HDLP active site, supporting the objective of improving linker group stability and aligning the KDACi within the hydrophobic tunnel of the “leg” and “exit” region. Based on adoption of this “heel-leg-exit” conformation, the predicted binding affinities of **3–7** for HDLP, determined via SPROUT, were good. For comparison, docking was also performed using AutoDock 3.0, as an independent check, utilising the autogrid utility, with docking guided by the grid map performed using the autodock tool; with fifty conformations generated per docked compound^76^. The most favourable binding energy (ΔG_bind_) of the docked conformation ranged from −13.0 to −14.0 kcal/mol, indicating a slightly higher affinity for HDLP than Entinostat (**1**) at −11.6 kcal/mol (Tables 1 and 2). The predicted increased binding affinity of **3–7** was indicative of their greater potential as class I selective KDACi over Entinostat (**1**), due to the proposed mechanism of action involving tighter enzyme binding and slower kinetics^77^^,^^78^. However, caution was adopted regarding indications of greater affinity for **3–7** over Entinostat (**1**), due to the well-known approximations that are necessary when modelling the binding of small molecules to proteins (e.g. treating the protein as a rigid structure and exclusion of water from modelling predictions^79^). Thus, confirmation of the potency of putative KDACi, relative to known compounds, was assessed in this study using various biological assays, as detailed below.

**Table 2.** Activity of putative class I selective KDACi.

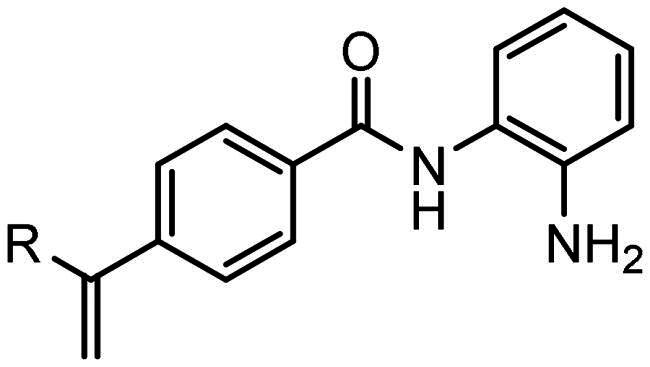								
Compound	R Group	ΔG_bind_ (kcal/mol)	pLogD_7.4_	HDAC Inhibition (µM)		A2780 IC_50_ (µM)	MCF7 IC_50_ (µM)	AC16 IC_50_ (µM)
				IC_50_	IC_20_			
**3**	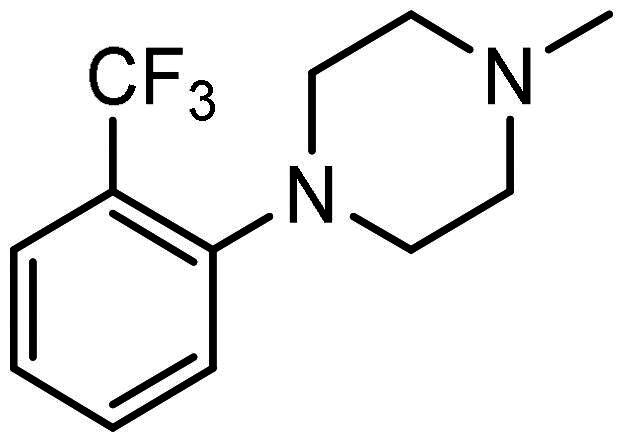	−13.6	4.67	>20	1.5 ± 0.8	7.5 ± 0.7	7.3 ± 0.4	>10
**4**	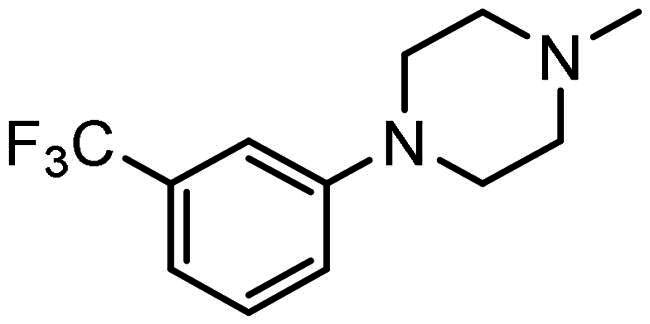	−13.3	4.68	>20	5.0 ± 1.5	1.6 ± 0.5	1.4 ± 0.1	>10
**5**	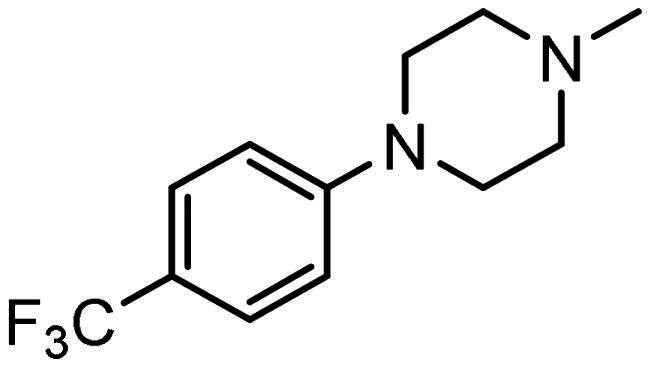	−13.8	4.66	>20	5.8 ± 1.8	1.9 ± 0.4	0.4 ± 0.4	>10
**6**	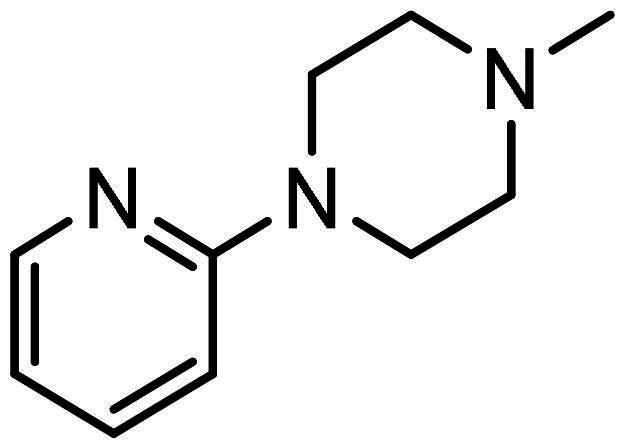	−13.0	2.48	>20	>10	3.5 ± 1.3	3.2 ± 0.8	>10
**7**	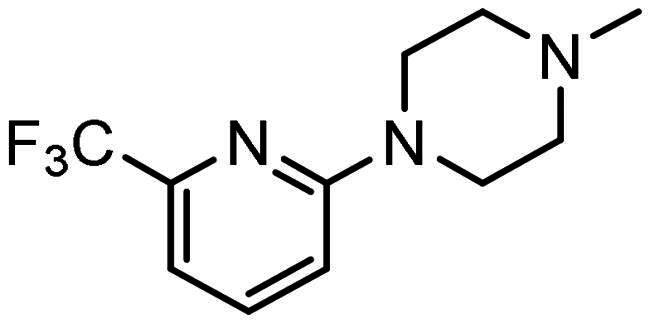	−14.0	4.03	>20	0.8 ± 0.2	0.8 ± 0.1	0.8 ± 0.3	>10

Inhibition of HDAC activity (as determined by Fluor-de-Lys assay), calculated binding energy (ΔG_bind_) by Autodock 3.0 of compounds against HDLP, pLogD_7.4_ values obtained from ChemSpider (http://www.chemspider.com), generated using the ACD/Labs Percepta platform-Physchem module, and antiproliferative activity against human A2780 ovarian and MCF7 breast cancer and AC16 cardiac cell lines.

All compounds were synthesised using a three-component catalytic coupling chemistry previously developed by our group^49^^,^^50^, involving a combination of the relevant nucleophile, tris(dibenzylideneacetone)dipalladium(0), tris(2-furyl)phosphine and base with a solution of an appropriate aryl halide followed by the introduction of allene gas, heating and subsequent purification (Figure 1).

### Predicted physiochemical, ADME properties, and drug-likeness

Evaluation of the Lipinski’s rule of five showed that compounds **3–7** had molecular weights between 413.5 and 481.5, three hydrogen bond donors, and five to six hydrogen bond acceptors. Compounds **3–5** exhibited LogP values of 5.1, slightly above the defined criterion of 5.0, with **6** and **7** fulfilling the requirements with LogP values of 3.3 and 4.3, respectively (Supplementary Table S2). In terms of Veber’s role, which established compounds with a polar surface area (TPSA) less than 140Å^2^ and 12 or less rotatable bonds (nRotB) are likely to exhibit good oral bioavailability, all compounds were identified as fulfilling these criteria and thus good oral bioavailability (supplementary Table S2). Therefore, the *in-silico* analysis of the physiochemical profiles of **1–7** confirms good potential as drugs, with compounds **6** and **7** showing the best drug-like properties based on their predicted physiochemical profile.

The predicted ADME profiles of the newly synthesised compounds **3–7** showed they had LogS values in water ranging from −4.82 to −6.11 and in buffer (pH 7.4) ranging from −5.85 to −6.59, indicative of moderate to low solubilities (Supplementary Table S3) and LogD_7.4_ values between 2.5 and 4.8 reflective of higher lipophilicity (Table 2). This contrasts the reference compound entinostat (**1**) with good aqueous solubilities (LogS of −3.81 and −3.17, respectively; Supplementary Table S2) and lower lipophilicity, LogD_7.4_ 1.92 (Table 1). All compounds demonstrated high predicted values for human intestinal absorption (HIA) of >90%, which alongside their higher lipohilicity (logD_7.4_) and satisfaction of Veber’s rules (Supplementary Table S1) further supports their potential as drug molecules.

In the context of biotransformation and excretion, the study found that **3–7** were predicted substrates for CYP3A4 and CYP2D6, whereas the reference compound entinostat (**1**) was a substrate for CYP3A4 but not CYP2D6 (Supplementary Table S2). Entinostat (**1**) was however predicted to inhibit the action of the major drug metabolising CYPs 3A4, 1A2, 2C19 and 2C9, in contrast to compounds **3, 4** and **7** which were not predicted as inhibitory (Supplementary Figure S2). Since CYP inhibition is a significant mechanism of drug-drug interactions, a significant clinical pharmacological liability^80^, this would suggest that these novel compounds, particularly compound **7**, have less of an adverse pharmacological risk than **1** (Supplementary Table S2). A key pharmacokinetic parameter within drug discovery is total clearance (CL_tot_), reflective of the overall ability of the body to eliminate the substance. The predicted log CL_tot_ values for **3–7** ranged from 0.56 to 0.87, indicating more rapid excretion than the reference compound **1** (log CL_tot_ of 0.14) (Supplementary Table S2). Taken together. These data suggest favourable ADME profiles of these putative KDACi, reinforcing their potential as viable drug candidates.

A major cause of drug attrition in the clinic is off-target toxicity, accounting for almost 40% of all cases, with adverse cardiac effects being a major cause^81^^,^^82^. Cardiac liability is commonly evaluated preclinically by a compounds ability to hinder the activity of the hERG cardiac potassium channel^82^. Low risk of hERG inhibition was predicted for entinostat (**1**) and compounds **6** and **7**, whereas a potential risk was identified for compounds **3–5** (Supplementary Table S2). This implies caution should be adopted should **3–5** be progressed clinically, conversely with **6** or **7** expected to exhibit low hERG-inhibitory cardiac liabilities.

### Compound-induced inhibition of cancer cell growth in vitro

All compounds **3–7** induced cytotoxicity at a concentration <10 µM against human ovarian A2780 and breast MCF7 cancer cell lines (Tables 1 and 2). In contrast, no significant cytotoxicity was detected with **3–7** against the normal human AC16 ventricular cardiac cell line (Table 2). In compounds with a trifluoromethyl-substituted aromatic ring, **3–5**, changing the substitution position gave rise to varying degrees of cytotoxicity against the cancer cell lines (Table 2). The presence of the trifluoromethyl group at the ortho position of the ring in **3** resulted in the least cytotoxicity, with values similar to that demonstrated for the non-selective and capless KDACi, Tacedinaline (**2**) (Tables 1 and 2). Moving the trifluoromethyl group from the ortho (**3)** to either the meta (**4**) or para (**5**) position resulted in a 4–5-fold increase in cytotoxicity (Table 2), achieving low micromolar cytotoxicity approaching that of the reference compound Entinostat (**1**) (Table 1). The meta-substituted compound (**4**) demonstrated the best cytotoxic profile of these particular compounds (Table 2).

Modification of the terminal capping group to a pyridine moiety (**6**), analogous to the reference compound Entinostat (**1**) and several more recently reported compounds, resulted in a slightly diminished cytotoxicity relative to **4** (Table 2). In an attempt to harness the binding interactions present for **1** and build upon the improved cytotoxicity observed with the 3-trifluoromethyl-substituted aromatic ring present in **4**, we subsequently synthesised a compound incorporating a 2,6-di-substituted pyridine as the terminal capping group (**7**). The introduction of this 6-trifluoromethyl-substituted pyridine significantly improved the compounds cytotoxicity profile relative to the unsubstituted pyridine (**6**) by approximately 5-fold. Furthermore, the activity of **7** was found to be improved relative to **4** and comparable to the sub-micromolar activity of Entinostat (**1**) (Tables 1 and 2).

### Compound-induced inhibition of histone acetylation in vitro

The KDAC inhibitory activities of **3–7** and the reference compound Entinostat **1** were evaluated against HeLa nuclear KDACs (Tables 1 and 2, Supplementary Figure S2). Interestingly, **3–7** did not exhibit an ability to inhibit enzyme activity by at least 50% (IC_50_), up to the maximal tested concentration of 20 µM (Tables 1 and 2, Supplementary Figure S2). It was hoped that this lack of detectable activity may be due to selectivity of compounds for specific class I KDACs within the nuclear cell extract, as well as compound lipophilicity and aqueous solubility, plus the relative insensitivity of the assay itself^83^. Consequently, for the purposes of this study, indicative KDAC inhibitory activity was recorded as the compound concentration required to inhibit HDAC activity by 20% (IC_20_; Supplementary Figure S2). All compounds evaluated, with the exception of the unsubstituted pyridine-capped compound **6**, exhibited KDAC inhibition IC_20_ values of <10 µM (Table 2, Supplementary Figure S2). Inhibitory activity of **7**, with the 6-substituted pyridine as the terminal capping group, was the most potent, with a KDAC inhibitory IC_20_ <1 µM (Table 2, Supplementary Figure S2). These results thereby supported the KDAC inhibitory potential of these compounds.

As indicated above, a major impact upon the KDAC inhibitory values identified for compounds **3–7** relative to **1** using the cell-free *in vitro* assay is likely to be their lipophilicity and reduced aqueous solubility, as previously discussed (Tables 1 and 2 and supplementary Table S2). To address this potential limitation within the assay, the hydrochloric salts, **5a** and **7a** were prepared (Table 3). With respect to enzyme inhibition, **7a** achieved a KDAC inhibition IC_50_ value comparable to the reference compounds **1** and **2** (Tables 1 and 3). These data substantiated the impact of lipophilicity and kinetic aqueous solubility on assay accuracy. It is however noteworthy that although higher lipophilicity may hinder the identification of the full inhibitory potential of these compounds using this cell-free *in vitro* assay, this parameter often shows a positive correlation with permeability and absorption, as previously discussed, important clinical parameters for drug development^84^.

**Table 3.** Activity of KDACi salts.

Compound	R Group	HDAC Inhibition (µM)		A2780 IC_50_ (µM)	MCF7 IC_50_ (µM)
		IC_50_	IC_20_		
**5a**	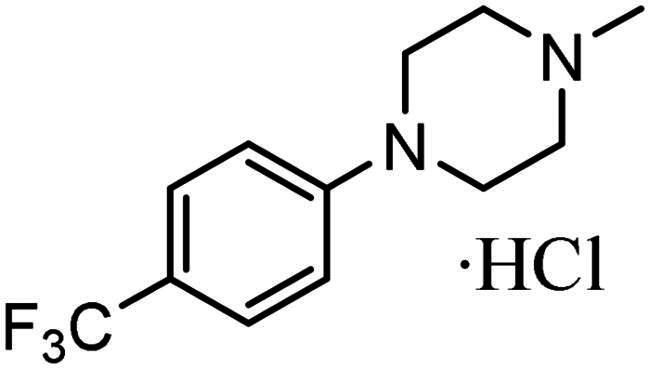	>20 *(>20)*	4.6 ± 3.4 *(5.8 ± 1.8)*	1.7 ± 0.2 *(1.9 ± 0.4)*	0.4 ± 0.2 *(0.4 ± 0.4)*
**7a**	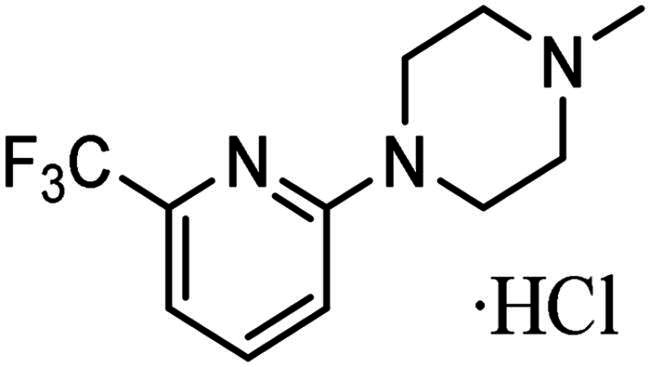	7.6 ± 2.5 *(>20)*	1.2 ± 0.4 *(0.8 ± 0.2)*	0.6 ± 0.04 *(0.8 ± 0.06)*	0.5 ± 0.2 *(0.8 ± 0.3)*

Inhibition of HDAC activity (as determined by Fluor-de-Lys assay) and antiproliferative activity against human A2780 ovarian and MCF7 breast cancer cell lines. Values in parentheses are those of the original compound.

The deacetylase inhibition values obtained for **3**–**7** (and to an extent **1** and **2**) may also be reflective of the mode of binding attributed to this class of compound. Benzamides have been demonstrated to bind in a two-step slow-on/slow-off “induced fit” mechanism against class I KDACs, associated with a delay in reaching equilibrium^77^^,^^78^. This mode of binding is not accounted for in these cell-free assays, likely resulting in an underestimation of KDACi capacity. A slow-binding “induced-fit” mechanism with low dissociation rates would inevitably relate to extended KDAC residency of compounds and subsequent higher inhibitor potency. Recent reports have demonstrated improvements in KDACi potency through the adoption of a prolonged enzyme-inhibitor pre-incubation step, substantiating this hypothesis^78^^,^^85^. Nevertheless, the data produced herein supported the ability of **3–7** to inhibit KDAC activity comparable to **1** and **2,** albeit with an indicative potency potentially lower than reality.

**Figure 3.**
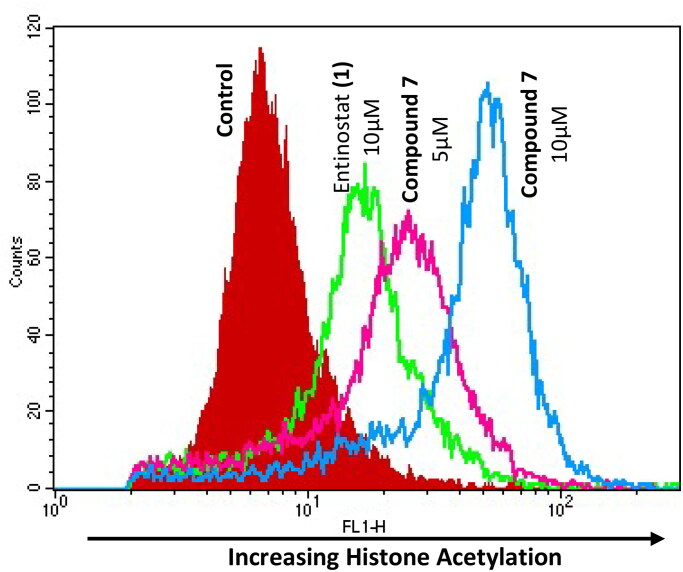
Increased induction in histone H4 acetylation by compound 7 compared to reference compound entinostat (1). Flow cytometric histogram depicting relative increase of acetylated histone H4 following a 24-h exposure of the A2780 ovarian cell line to either 5 µM or 10 µM of the respective KDACi.

Evaluation of the ability of these compounds to affect histone H4 and tubulin acetylation *in situ* within cells was assessed, using our previously reported flow cytometric methodology^68^. Increased acetylated histone H4, a target of class I KDACs, was observed with both **1** and **7** (Figure 3). In contrast, neither **1** nor **7** induced a measurable change in tubulin acetylation (Supplementary Figure S3), a marker of class II KDAC activity, reinforcing the selectivity of these compounds towards class I KDACs (HDAC 1–3). This was further confirmed by the time-dependent increase in histone H4 hyperacetylation observed with **7** by western blotting (Supplementary Figure S4). The degree of induction of histone H4 acetylation, determined by sensitive flow cytometry, was observed to be much greater for **7** relative to **1** at an equimolar concentration, indicative of potential differential KDAC selectivity between these two compounds (Figure 3). Together these data strongly reinforce the selectivity of these compounds towards class I KDACs.

### Antiproliferative activity profile of KDACi against different tumour types in vitro

A lack of clear similarity in cytotoxicity profiles against the 60-cell line panel (NCI-60)^63^ were identified between compounds **3–7** and Entinostat (**1**), evidenced both between and across tumour types (Table 4), indicative of either differential potencies against the same target (presumably class I KDACs) or selectivities between KDAC isoforms. This opens up the value and scope for the development of KDACi with targeting for particular tumour types.

**Table 4.** Differential cytotoxicity IC_50_ values of compounds against NCI-60 tumour cell-line panel.

	Compound					
	1 *(entinostat)*	3	4	5	6	7
Leukaemia						
HL-60	0.85	0.04	0.12	0.08	2.19	0.32
K562	0.51	1.54	0.18	0.30	0.96	–
MOLT-4	1.93	0.13	0.13	0.60	–	0.86
RPMI-8226	0.90	0.16	0.32	0.40	1.60	0.53
SR	0.53	2.36	0.02	1.10	5.33	0.28
NSCLC						
A549	3.28	4.17	1.48	6.43	2.39	3.32
EKVX	3.26	6.70	2.92	12.36	5.33	24.27
HOP-62	2.06	4.76	0.70	8.38	1.32	2.21
HOP-92	0.04	1.85	1.51	3.94	5.70	–
H226	0.60	7.78	1.89	7.52	–	13.43
H23	3.22	5.93	3.72	9.48	0.94	14.09
H322M	4.79	11.40	0.94	40.46	3.12	>100
H460	0.83	5.58	1.56	3.44	2.81	0.64
H522	0.70	1.91	6.14	1.85	1.56	1.11
Colon						
COLO205	2.00	11.46	3.45	>100	1.71	3.33
HCT116	0.80	3.09	1.14	11.38	2.50	0.54
HCT15	8.47	4.89	0.48	>100	1.91	0.92
HT29	1.03	4.09	1.46	75.68	–	0.56
KM12	4.31	2.04	0.49	7.64	0.94	0.67
SW620	1.30	3.40	1.28	78.89	1.71	0.53
CNS						
SF-268	1.86	4.55	1.43	6.78	1.66	1.12
SF-295	2.56	5.97	0.54	10.79	1.39	0.39
SF-539	3.19	3.27	0.54	8.15	1.50	0.98
SNB-19	6.34	24.37	4.10	12.56	4.86	0.65
SNB-75	5.07	2.82	0.01	12.45	1.48	0.67
U251	2.03	4.63	1.17	6.25	1.48	1.16
Gastrointestinal						
OVCAR-5	1.05	11.83	6.62	>100	6.10	0.71
Melanoma						
LOX IMVI	1.06	5.86	0.12	11.09	0.93	1.49
MALME-3M	0.58	4.18	–	19.28	–	1.06
M14	2.35	4.47	1.05	90.57	8.47	0.97
MDA-MB-435	0.96	2.72	0.14	25.18	0.53	0.27
SK-MEL-2	1.10	10.28	3.44	>100	1.87	5.65
SK-MEL-28	0.66	10.35	3.40	>100	2.52	2.08
SK-MEL-5	0.74	3.01	0.57	4.43	0.73	0.40
UACC-257	1.43	4.67	0.76	2.39	2.32	26.54
UACC-62	0.28	9.16	0.41	22.96	1.41	0.97
Ovarian						
IGROV-1	3.95	6.03	14.19	18.24	1.57	2.01
OVCAR-3	1.74	5.92	0.55	11.99	1.24	0.26
OVCAR-4	1.31	6.53	3.94	5.68	6.35	8.36
OVCAR-8	1.03	3.84	1.40	6.95	1.50	4.25
NCI/ADR-RES	2.38	1.87	0.11	3.37	1.31	0.64
SK-OV-3	2.75	10.96	–	12.13	1.30	2.55
A2780*	*0.6*	*7.50*	*1.60*	*1.90*	*3.50*	*0.8*
Renal						
786-0	4.97	2.36	1.10	2.85	1.21	1.97
A498	0.36	3.55	0.01	7.67	–	0.97
ACHN	2.33	7.19	0.92	9.64	1.50	8.39
CAKI-1	1.17	7.96	0.63	6.08	1.19	0.51
RXF 393	1.49	1.67	1.62	1.97	1.55	0.67
SN12C	9.40	6.43	0.29	6.37	2.36	3.79
TK-10	0.29	4.63	0.81	20.99	1.85	16.41
UO-31	1.17	1.00	2.39	0.97	1.80	2.75
Prostate						
PC-3	2.79	1.91	0.62	1.29	3.78	0.42
DU-145	2.26	7.52	1.36	20.99	2.52	1.45
Breast						
MCF-7	0.64	4.48	1.24	10.89	3.16	0.35
MDA-MB-231	13.8	4.68	0.94	3.60	2.17	4.62
HS 578 T	2.33	12.77	0.49	22.54	1.11	0.45
BT 549	4.14	2.67	0.33	6.92	3.94	0.92
T-47D	0.46	7.67	13.2	21.83	1.23	3.01

**Table:** 

IC_50_ value	
<1 µM	
1–5 µM	
5–10 µM	
10–100 µM	
>100 µM	

All compounds (with the exception of **6**) exhibited a high level of cytotoxicity against leukaemic cell lines, a response reflective of a central role for KDACs in the development and progression of haematopoietic malignancies^86^^,^^87^. Interestingly, compounds **3** and **5** were particularly selective towards leukaemias, with nanomolar cytotoxicities against this tumour type relative to much higher micromolar activities against other tumour types (Table 4). This suggests that the precise nature of the capping group is important for leukaemic activity, as the presence of the trifluoromethyl group at the ortho (**3**) or para (**5**), but not the meta (**4**), position of the aromatic ring delivers anti-leukaemic selectivity.

Against solid tumours, a range of intriguing differences in cytotoxicity profiles were observed compared to the reference compound Entinostat (**1**) (Table 4). Activity against solid tumours was afforded by the incorporation of a trifluoromethyl group at the meta- position of the aromatic capping group (**4**) (Table 4). Modification of the aromatic ring of **4** to a pyridine incorporating the trifluoromethyl group at the analogous 6-position (**7**) retained- and in many cases improved the cytotoxicity of these KDACi (Table 4), in agreement with the initial cytotoxicity screen (Table 2). The importance of moieties on the capping group, in this case a trifluoromethyl group, is illustrated by the limited cytotoxicity exhibited by **6** with its unsubstituted pyridine capping group (Tables 2 and 4).

The majority of tumour types evaluated within the NCI-60 screen suggested a greater cytotoxic response to **4** and **7** compared to **1** (Table 4), including colorectal, central nervous system (CNS), breast, ovarian, prostate and renal cancers. Against colorectal cancer cell lines, only one (of six) within the NCI-60 cell line panel exhibited nanomolar GI_50_ values against Entinostat **1** (Table 4), values comparable to previous *in vitro* studies^88^. In comparison **4**, with the trifluoromethyl group at the meta position of the aromatic ring, exhibited nanomolar cytotoxicities against two of the six colorectal cell lines evaluated (HCT15 and KM12), with further modification of the capping group from a substituted aromatic ring to a substituted pyridine (**7**) resulting in an improvement in cytotoxicity potential (Table 4). In the case of CNS tumours, Entinostat (**1**) did not induce cytotoxicity <1 µM in any of the six CNS cell lines tested, whereas **4** and **7** exhibited nanomolar cytotoxicity potencies in three and four out of six of these cell lines, respectively (Table 4). In this tumour type, cytotoxicity of **4** and **7** was often several fold greater than that of **1**, most notably against the highly aggressive SNB-75 glioblastoma cell line; **1** (5.07 µM), **4** (10 nM) and **7** (670 nM). This trend was also evident with the aggressive prostate PC-3 and aggressive breast HS578T and BT549 cell lines (Table 4).

Cell lines reflecting triple-negative breast cancer (MDA-MB-231, HS578T and BT549), representing the most challenging type of breast cancer, were poorly responsive to **1** but were all highly sensitive to **4**. Conversely, the non-aggressive T-47D breast cell line was sensitive to **1** but >6-fold less sensitive to **7** and >28-fold less sensitive to **4**. In the context of a drug-resistant phenotype, the HCT15 colorectal cell had been previously identified as being “resistant” to KDACi, including Entinostat (**1**), a claim upheld in this study (Table 4)^89^^,^^90^. However, this “resistance” of HCT15 to **1** and other KDACi was not observed with either **4**, **7** or the second generation class I KDACi mocetinostat (Table 4)^46^. This supports either a different and/or improved KDAC selectivity profile for **7** (and **4**) or an ability to circumvent “resistance” mechanisms evident for Entinostat (**1**). Taken together, these apparent relationships of **4** and **7** towards greater responses in more aggressive cancers is potentially exciting, especially since clinical management of such tumours is challenging.

Despite **4** and **7** exhibiting comparative cytotoxicity potentials in the majority of cases, with **7** having a slightly better profile than **4**, there are some distinct differences between responses to these KDACi. For instance, **4** exhibited approximately 100-, 35- and 20-fold improvement in cytotoxicity sensitivity against the H322M NSCLC, UACC-257 melanoma and TK-10 renal cell lines compared to **7** (Table 4). Conversely, against the OVCAR-5 gastrointestinal tumour cell line, **7** was shown to be 10-fold more efficacious than **4** (Table 4). This is very intriguing as the only difference between these compounds is the additional nitrogen in the pyridine capping group of **7.** Potential reasons for these differences include minor differences in lipophilicity and cellular uptake, or variations in KDAC isotype affinities. However, a poly-pharmacological activity of these compounds may also exist, with additional secondary off-target activities, synergistic or additive to their KDACi role. This theory is supported by the class I selective benzamide KDACi chidamide, which alongside inhibition of KDACs also inhibits nicotinamide phosphorribosyltransferase (NAMPT) to evoke its antitumour activity^91^^,^^92^. As such, there is now increasing evidence supportive of a multi-targeted polypharmacological approach of aminobenzamide-based KDACi^92^. Such an activity offers an explanation for differential efficacies of **4** and **7** versus **1** against solid and drug-resistant tumour types. Further studies are thereby warranted to clarify the potential therapeutic advantages of these compounds.

### Identification of novel mechanisms of action of KDACi via COMPARE analysis

COMPARE analysis showed that **3–7** poorly correlated with the reference compound Entinostat (**1**), with all correlations having *ρ* < 0.2 (Table 5). When compared to those within the NCI/NIH DTP database, **1** showed a strong correlation in activity (*ρ* > 0.7) to only two compounds, the second-generation class I selective KDACi mocetinostat (NSC 760143; *ρ* = 0.77), and the pan-phosphoinositide-3-kinase (PI3K) inhibitor Buparlisib (NSC75435; *ρ* = 0.70). No significant cytotoxicity associations were observed between **1** and any other KDACi within the database. With regards to **3–7**, correlations to Mocetinostat were weak with *ρ* ranging from 0.12 with **5** to 0.33 with **7** (Supplementary Table S4).

**Table 5.** Similarity index of antitumour activity of compounds screened against the NCI-60 tumour cell line panel derived through use of the COMPARE algorithm.

Compound	1	3	4	5	6	7
1						
3	0.13					
4	0.02	0.37				
5	0.08	0.80	0.31			
6	−0.04	0.10	0.20	0.06		
7	0.15	0.32	0.35	0.18	0.09	

Correlations recorded as the matrix COMPARE coefficients (*ρ* values), with *ρ* value >0.7 deemed significant (indicated in yellow in table) and with *ρ* value <0.7 deemed non-significant.

Within this compound series, a strong correlation (*ρ* = 0.80) was identified between **3** and **5**, which exhibit exquisite selective activity against leukaemic cell lines and potentially the same mechanism of action and KDAC selectivity profile (Table 4). Conversely, **4** and **7**, which showed the greatest profile of cytotoxicity activities against solid tumours (Table 4) and only differed due to the presence of the additional nitrogen in the pyridine capping group of **7,** did not demonstrate a strong correlation (*ρ* = 0.35) to each other. This indicates that despite both exhibiting potent cytotoxicity, these compounds may exhibit differential selectivity for specific class I KDACs or poly-pharmacological actions, but more importantly reiterates the significant impact of the orientation and structure of the KDACi capping group.

In terms of identifying relationships to modes of action based on compounds in the database, none of the compounds **3–7** exhibited relationships to any compound in the database with a known and confirmed mechanism of action (Supplementary Table S4). However, this does not preclude **3–7** acting primarily as a KDACi, as the majority of the comparative compounds from the database are novel compounds (as are the compounds in this study) for which a mechanism of action has not yet been identified. Furthermore, a lack of correlations to a widespread therapeutic mechanism is also not exclusionary, as is evident by the lack of correlation between the established clinical candidate **1** with other known KDACi within the database. Taken together, the fact that **3**–**7** did not indicate strong correlations in the database to other previously evaluated compounds, highlights their novelty and reinforces their potential scope as chemotherapeutic agents.

### Isotype-selective class I KDAC inhibition in vitro

Lead compound **7** inhibited activity of class I KDACs (HDAC1, HDAC2 and HDAC3), with selectivity noted for HDAC1 over HDAC2 and HDAC3 (Table 6). No activity was detected for **7** against any class IIa KDACs (HDAC4, 5, 7 or 9), class IIb KDACs (HDAC 6 and 10), class IV KDAC (HDAC11) or the class I KDAC family member HDAC8 (Table 6). The inhibitory activity of **7** against HDAC1 was comparable to that of Tacedinaline (**2**), which is devoid of a capping group (Table 6). However, the selectivity of **7** for HDAC1 over HDAC2 and HDAC3 contrasted with the non-selectivity of **2** and more aligned with that observed for Entinostat (**1**), supporting an important role for the capping group in delivering class I KDACi isotype selectivity. Inhibition of the class IIa KDAC HDAC9 by **1** but not by **7** offers an alternative explanation for the differential cytotoxicity profile of **1** relative to **7**, considering HDAC9 has recently been associated with promoting cancer cell survival^93^. However, the consequence of HDAC9 inhibition by **1** and the degree of involvement in its therapeutic activity have yet to be determined.

**Table 6.** Inhibitory potency (IC_50_) of compound 7 and reference compounds 1 and 2 against recombinant human HDACs.

	Compound IC_50_ (µM)										
	Class I				Class IIa				Class IIb		Class IV
Inhibitor	HDAC1	HDAC2	HDAC3	HDAC8	HDAC4	HDAC5	HDAC7	HDAC9	HDAC6	HDAC10	HDAC11
TSA	0.02	0.03	0.03	0.54	*ND*	*ND*	*ND*	*ND*	<0.01	0.04	3.65
TMP269	*ND*	*ND*	*ND*	*ND*	0.34	0.20	0.08	0.02	*ND*	*ND*	*ND*
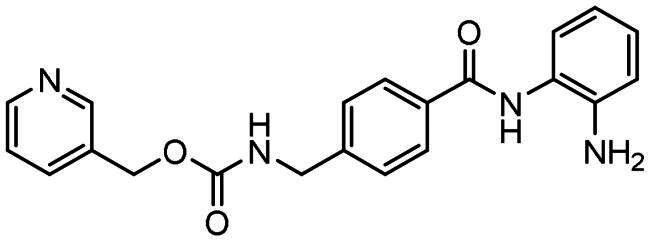 **1** (Entinostat)	0.18	1.15	2.31	>10 µM	>10 µM	>10 µM	>10 µM	0.51	>10 µM	>10 µM	*ND*
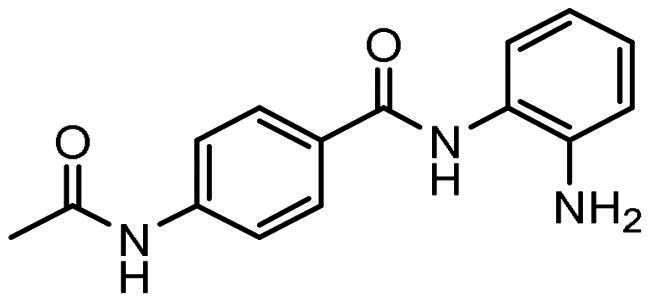 **2** Tacedinaline (CI-994)	0.90	0.90	1.20	>10 µM	>10 µM	>10 µM	>10 µM	>10 µM	>10 µM	>10 µM	>10 µM
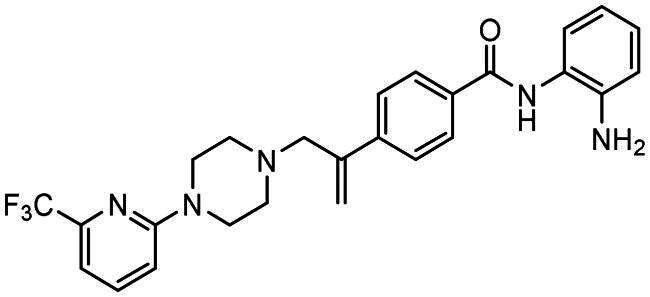 **7**	0.89	3.97	7.79	>10 µM	>10 µM	>10 µM	>10 µM	>10 µM	>10 µM	>10 µM	>10 µM

ND: not determined.

IC_50_ is the concentration that reduces initial HDAC enzyme activity to 50%.

Values for TSA, TMP269 and **6** were determined by Reaction Biology Screen Services

TMP269 is a HDAC class IIa selective inhibitor used as an internal standard for this assay.^100^

Data for compounds 1 and 2 is compiled from Ref. 13.

Although at face-value, these results using recombinant enzymes imply a greater affinity for HDACs 1–3 by **1** versus **7** (Table 6), the higher lipophilicity of **7** (pLogD_7.4_ = 4.03) relative to **1** (pLogD_7.4_ = 1.92) and consequent reduced aqueous solubility (supplementary Table 2) and low volume assay is a major factor in this context, as was the case with the initial cell-free KDACi assay (Table 1 and 2). Furthermore, the slow binding kinetics of these compounds to KDAC, a major contributory factor for their inhibitory capacity^78^^,^^94^, and higher predicted binding energy of **7** (ΔG_bind_ −14.0 kcal/mol) relative to **1** (ΔG_bind_ −11.6 kcal/mol) are also likely to be contributory factors to this differential. As such, the actual KDACi IC_50_ values for **7** (and potentially **3–6**) may be much lower than recorded. Notwithstanding this, these data confirm the ability of **7** to selectively inhibit class I KDACs, a mechanism strongly supported by pharmacodynamic studies indicating a superior ability of **7** compared to **1** to induce acetylation of histone H4 over an extended time-period (Figure 3, Supplementary Figure S4).

One further interesting observation is the disparity between KDAC inhibitory and cytotoxicity IC_50_ values (Tables 1–4 and 6). For instance, 7 inhibits HDAC1 at 900 nM but exhibits cytotoxicity IC50 values against leukemic cell lines between 280 and 860 nM (Tables 4 and 6). This reverse differential has also been reported for other benzamide-containing KDACi^91^^,^^95^. For instance, a series of potent analogues of the class I selective KDACi’s Chidamide and Entinostat, inhibited HDAC1 and HDAC2 with IC_50_ values of 218 nM and 407 nM, respectively, with cytotoxicity IC_50_ values against malignant cell lines of <100 nM^95^. This was shown to be a result of these compounds also inhibiting tubulin polymerisation alongside KDAC inhibition^95^. In the case of chidamide, which inhibits class I HDACs with IC_50_ values of 110–330 nM and induces cytotoxicity against leukaemic cell lines at concentrations below this, a mechanism involving dual inhibition of NAMPT and potent KDAC inhibition has been demonstrated^91^. Therefore, as discussed earlier, a multi-targeted action is becoming increasingly common for class I selective benzamide-based KDACi^92^. Although the beneficial nature of this polypharmacological activity is becoming increasingly evident, it remains to be determined whether this occurs with the compounds herein and which additional target(s) may be involved.

### X-ray crystal structure of KDACi

**Figure 4.**
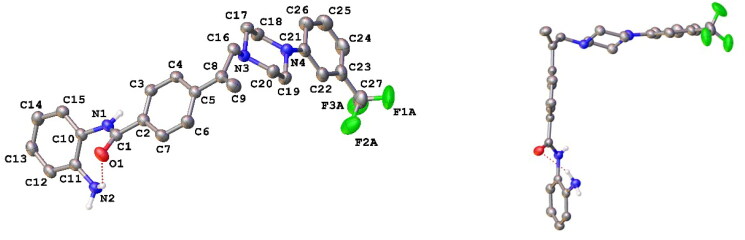
The asymmetric unit of the crystal structure of **4** with atomic numbering scheme (left) and in an orientation demonstrating a molecular “kink” in the linker region (right). Ellipsoids are drawn at the 50% probability level and hydrogen atoms bound to carbon atoms have been omitted for clarity.

Analysis of single crystals of **4** identified a conformation in the solid state with a pronounced “kink” of *ca*. 90° (Figure 4). The location of this “kink” is noteworthy as this arises due to the torsion about the C8-C16 bond (N5-C8-C16-N3 60.10(15)°). Another salient feature of the structure is that the double bond orientates itself into a near coplanar arrangement with the benzamide group, maintaining the resonance stabilisation of this extended system with a twist angle of *ca*. 22° observed between the [C9 C8 C16] plane and the adjacent benzene ring. The slight deviation from ideal planarity observed is attributable to steric interactions between the vinyl and aromatic hydrogen atoms. This structure was then used for *in silico* modelling to address binding into the enzymatic active site.

### Differential alignment of 3–7 relative to entinostat (1) within the class I KDAC active site

**Figure 5.**
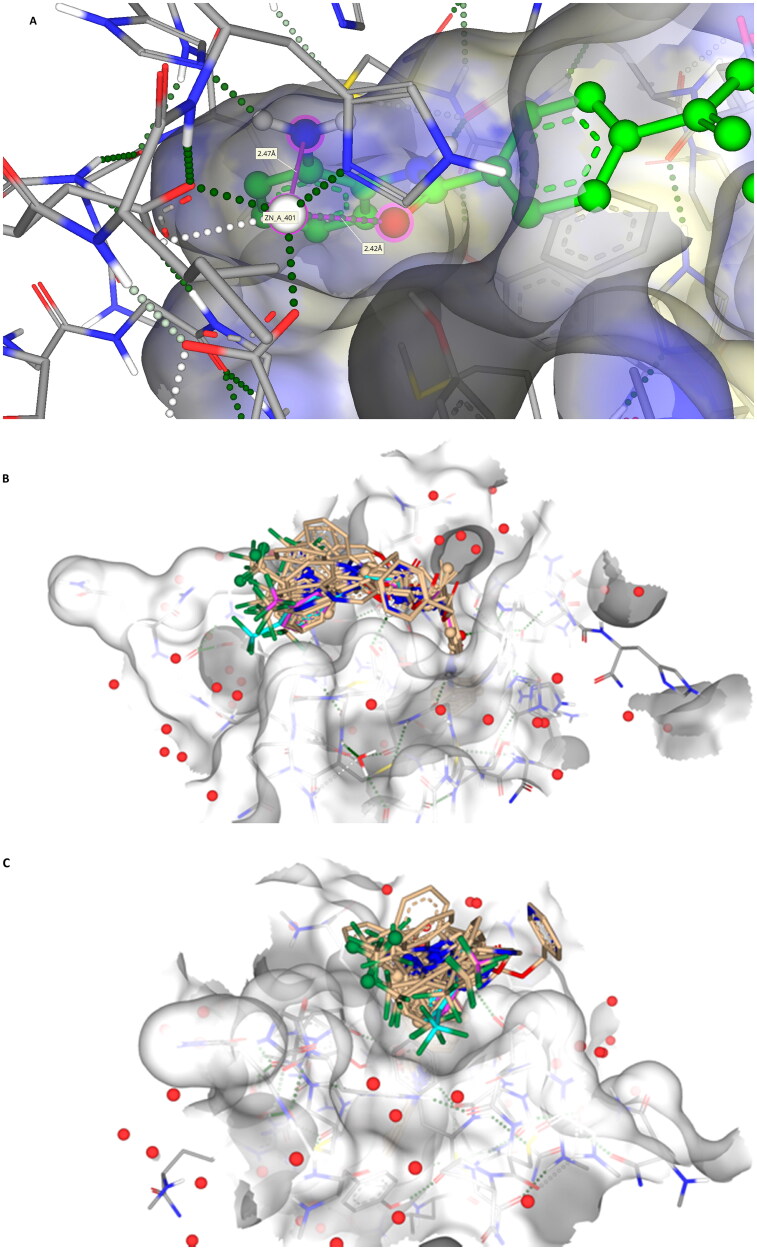
(A) The docking pose of **7** (green) highlighting the hydrogen bonds made to the zinc (coloured dashed lines) with binding of the benzamide headgroup of 7 to the zinc (bonding and distances shown); (B) Docking showing the surface binding (red dots indicate water molecules); (C) Head-on visualisation of the 34 poses showing the surface binding grove exploited by the compounds (red dots indicate water molecules).

Initial *in silico de novo* design of **3–7** employed the crystal structure of HDLP, co-crystalised with the pan-HDACi trichostatin^75^ (Figure 1). Although this structure was good for modelling binding in the “foot” and “leg” of the KDAC, it provided limited scope for the evaluation of interactions at the upper-end and external surface regions of the KDAC active site and did not fully recapitulate the human class I KDAC structure. Therefore, to better understand the binding mode of KDACi and their interactions with the “leg” and “exit” of the enzymatic cavity, modelling using specific mammalian KDACs was employed. To date, only HDAC2 has been co-crystallized with benzamide-containing inhibitors (PDB codes 3MAX, 4LY1, 5IWG, 5IX0 and 7KBG)^47^^,^^96^^,^^97^. We subsequently performed *in silico* docking studies using the crystal structure of human-HDAC2 (PDB: 7KBG) and the SeeSAR molecular docking programme^61^^,^^96^. Importation of **1**, **3–5** and **7** into SeeSAR predicted that the piperazine nitrogen closest to the vinyl group in **3–5** and **7** to be protonated at physiological pH, with the pKa at this position calculated to be 7.0. Following optimisation and triage, a total of 34 docking poses into HDAC2 were obtained for **1, 3–5** and **7**, with estimated binding affinities in the pM to nM range, very good ligand lipophilic efficiencies (LLE) and a mixed but positive torsion statistic profile. In all cases, this docking replicated the heel-foot binding conformation initially obtained using SPROUT (Figure 1), wherein zinc-binding occurred through the benzamide carbonyl moiety and the o-amine of the benzamide (distances C = O---Zn 2.42Å and N---Zn 2.47Å) whilst the amide nitrogen shows a binding interaction with the carbonyl of Gly-150 (Figure 5A). This binding mode within the 3D model is orientated to allow the linker to present the capping groups at the surface, exploiting a surface cleft in the protein (Figure 5B,C), with interactions in this region known to tune the selectivity and potency of KDACi.

**Figure 6.**
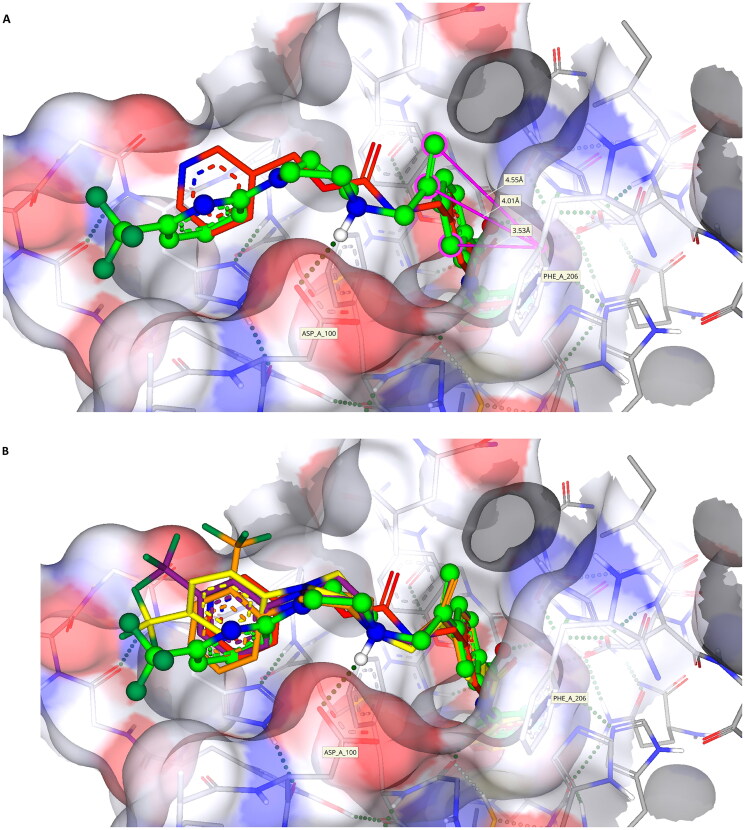
(A) Compound **1** (coloured red) and **7** (green) posed together to highlight the interaction of the vinyl and aromatic group with Phe-206 (pink lines/labels) and hydrogen bonding of the piperazine to Asp-100.[It should be noted that all analogues **3–5** show nearly identical interactions to **7**, they have been excluded for clarity]; (B) Compounds **1** (red), **3** (orange), **4** (purple), **5** (yellow) and **7** (green) showing the capping group orientations inside the cleft (green dashed lines show hydrogen bonding interactions).

Further comparative analysis of the predicted binding modes of KDACi **3–5** and **7** against **1**, demonstrated that the aromatic ring adjacent to the vinyl group in **3–5** and **7** is presented further up the “leg” in contrast to **1**. Focusing on our lead compound **7**, our model presents the vinyl group close to the Phe-206 residue, implying a degree of stabilisation from π-π stacking interactions when compared to **1** where no such interaction is possible (Figure 6A). Moreover, the aforementioned “kink” observed in the solid form of the molecule positions the capping group into the cleft at the exit of the enzymatic active site. This positions the protonated piperazine nitrogen of **7**, near to the Asp-100 residue, allowing for a strong hydrogen bond to be formed. This binding pattern is replicated with compounds **3–5**. In contrast, whilst **1** has a nitrogen moiety nearby, it is not close enough to establish a stabilising interaction with Asp-100, further disadvantaging its binding affinity (Figure 6A). Taken together, this supports the more favourable positioning within the enzyme active site of **3–7** relative to **1**, allowing improved alignment of the molecules into the KDAC-discriminatory cleft at the exit of the active site (Figure 6B).

**Figure 7.**
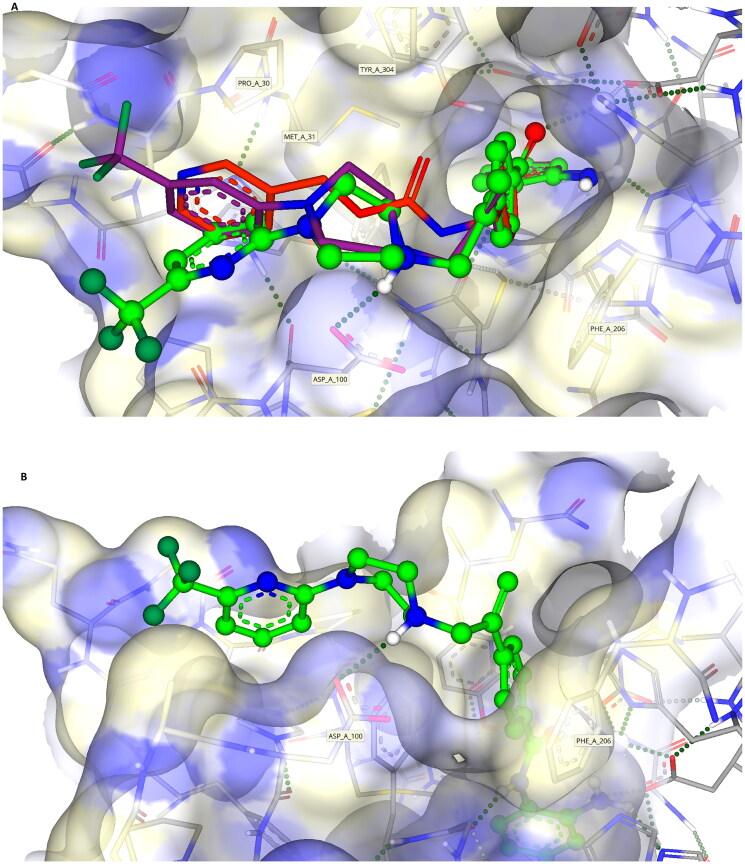
(A) Image showing compounds **1** (red), **4** (purple) and **7** (green) bound with **7** being on the more hydrophilic side of the cleft (pointing towards the Asp-100 residue, blue surface) whilst **1** and **4** orientate to the more hydrophobic side (proline, yellow surface) of the cleft; (B) Compound **7** (green) bound to the more hydrophilic side of the surface cleft;.

In addition to predicted differences in binding configuration between **1** and **3–7** within HDAC2, there are significant differences in the orientation between compounds **3–7** in the cleft, a highly interesting observation because of the close similarities in their structure. This variation in the docking of **3–5** versus **7** is attributed to the positioning of the substituted aromatic moiety of their respective capping groups into the cleft at the exit of the active site of HDAC2. A comparison between **4** and **7**, in which the only difference is the presence of nitrogen in the terminal capping group, exemplifies this. The capping group of **7** locates primarily towards the hydrophilic side of the cleft, whereas **4** shows the opposite tendency and exhibits a preference for the opposing, more lipophilic face of the cleft (Figure 7A). The positioning of **4** in the cleft is analogous to that of Entinostat (**1)**, further supporting the differential between **1** and **7**. One potential reason for the difference in alignment is the contrasting electronegativities, with the more electronegative pyridine ring (**7**) favouring the more hydrophilic face of the cleft at the active site exit (Figure 7B). Taken together such a difference offers significant scope for the design and development of improved KDACi selective compounds.

### Differences in the positioning of KDACi 7 in the active site of HDAC1 versus HDAC2

The docking of **7** into HDAC2 and its alignment provides a good indication of the relative positioning of these compounds in class I KDACs (Figure 7), building upon initial studies using HDLP (Figure 1). However, despite close structural homology between the class I KDAC family members, a clear differential in KDAC inhibitory activity is shown with **7** against these two enzymes, with inhibition of HDAC1 being much greater than HDAC2 (Table 6). This observation is indicative of differences in affinity or residency of **7** in HDAC1 versus HDAC2.

However, for *in silico* studies, unlike HDAC2, there are no current models in which HDAC1 has been co-crystalised with a benzamide-containing KDACi. Consequently, to identify the binding region and surface-based residues of HDAC1 we utilised the HDAC1 crystal structure (PDB: 4BKX, apo-form) and SeeSARs binding site module. This revealed a binding pocket with a Zn(II) atom and the familiar “foot-heel-toe” shape and a similar surface area and volume to HDAC2. As previously undertaken, **7** was docked into this model utilising the same criteria as outlined for HDAC2, with poses obtained showing compelling and comparative interactions with the protein.

**Figure 8.**
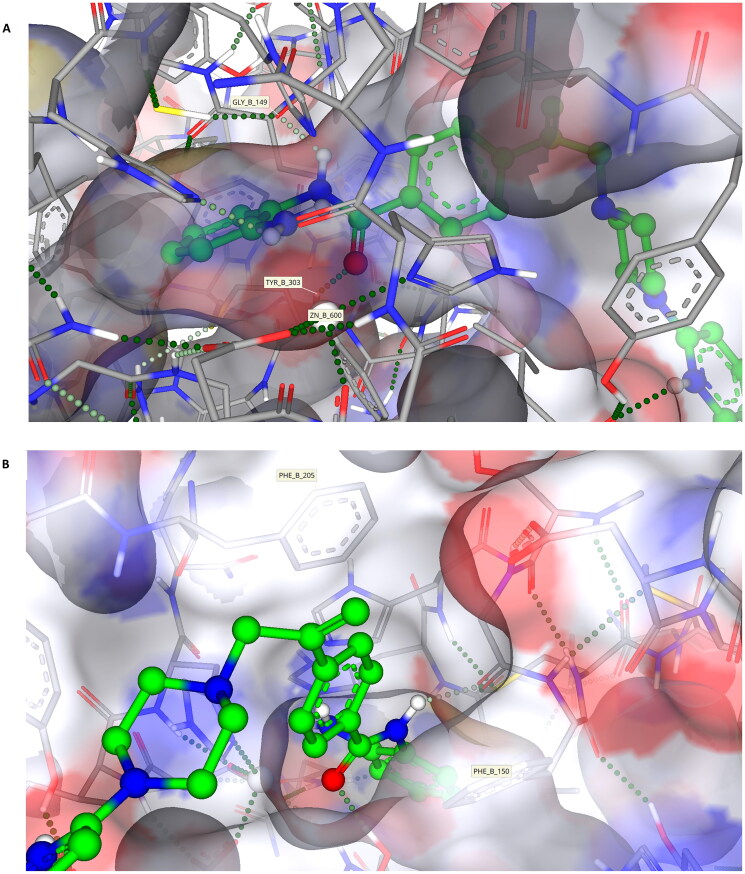

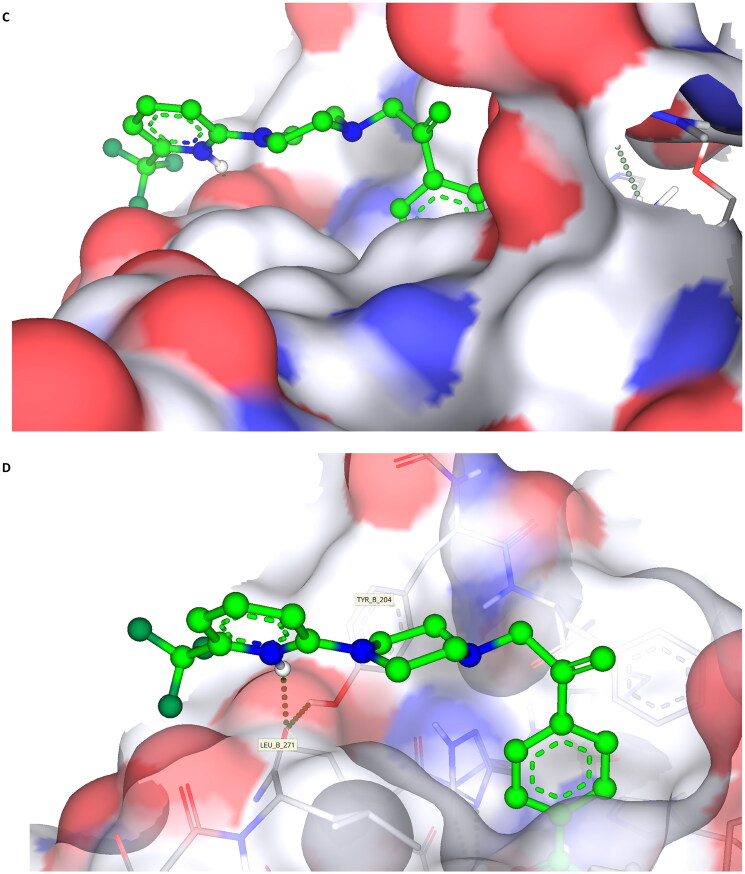
(A) Model showing the docking of **7** (green) into the active site of HDAC1 (PDB:4BKX) showing interactions (green dash hydrogen bonds) with Tyr-303 and Gly-149 and the restricted nature of the binding “foot-leg” pocket; (B) Model showing the extension of the vinyl group out of the “leg” binding pocket and the position of the two residues Phe-150 and Phe-205 sandwiching the aromatic linker of **7** (green); (C) Model looking down the previously identified surface cleft of HDAC1 towards compound **7** (green); (D) Model showing the conformation of the piperazine ring and interaction with Leu-271 of compound **7** (green).

Key residues previously reported for the HDAC1 active site involve binding of benzamide-containing KDACi with interactions between the carbonyl oxygen of the benzamide and the tyrosine residue, and the amide interacting with the glycine residues within the active site^98^, both of which were evident in our model of the binding of **7** and HDAC2 (Figure 5, 7 and 8).

The vinyl group of **7** appears to play less of a role in binding within HDAC1 than HDAC2, with this group extending further out of the active site rather than into the “leg” portion of the enzyme, suggesting the zinc is further down the “leg” in HDAC1 relative to HDAC2. This places the aromatic ring of the “linker” portion of **7** close to the Phe-205 residue (comparable to Phe-206 in HDAC2) and also Phe-150 with both appearing to pinch the aromatic ring providing two strong π-π stacking interactions in stark contrast to HDAC2 (Figure 8).

Whilst the vinyl group is further out of the enzymatic “leg” cavity in HDAC1 relative to HDAC2, it continues to “kink” the molecule which we believe is crucial to the placement of the capping group onto the protein surface. As discussed earlier, the vinyl group will remain near coplanar with the aromatic system and in doing so orientates the capping group on the protein surface away from a significant cleft in the protein, previously identified as a binding region for benzamide inhibitors (Figure 8C)^99^. Nevertheless, the identified clefts in the surface region possess several unique features between HDAC1 and HDAC2 that compound **7** can take advantage of, namely the hydrophobicity, openness and position of important binding residues. This is supported by the SeeSAR prediction, which indicated the hydrophobic nature of the HDAC1 surface would maintain the piperazine in a deprotonated state, in contrast to HDAC2 where it is protonated, permitting a greater degree of surface binding of HDAC1. Additionally, when bound into HDAC1, the piperazine ring of **7** is predicted to adopt the more stable chair conformation, whereas in HDAC2 it is predicted to adopt a less stable twisted boat conformation, adding additional strain to the molecule and reducing its interactions and potential active site residency in HDAC2 compared to HDAC1. However, the most significant difference between HDAC1 and HDAC2 in this region, which may account for the increased inhibitory activity of **7** against HDAC1 versus HDAC2, is the availability of a hydrophobic leucine residue (Leu271) in HDAC1. This amino acid makes a strong hydrogen bonding interaction with the pyridine nitrogen at the terminus of the capping group of **7**, a stark contrast to the much weaker electrostatic interactions observed with the hydrophilic cleft surface in HDAC2 (Figure 8D). Taken together, the unique structural features of **7** and differences in the protein surface between HDAC1 and HDAC2, support the selectivity of this compound for HDAC1 over HDAC2.

### Induction of antitumour activity against human tumour xenograft in vivo

**Figure 9.**
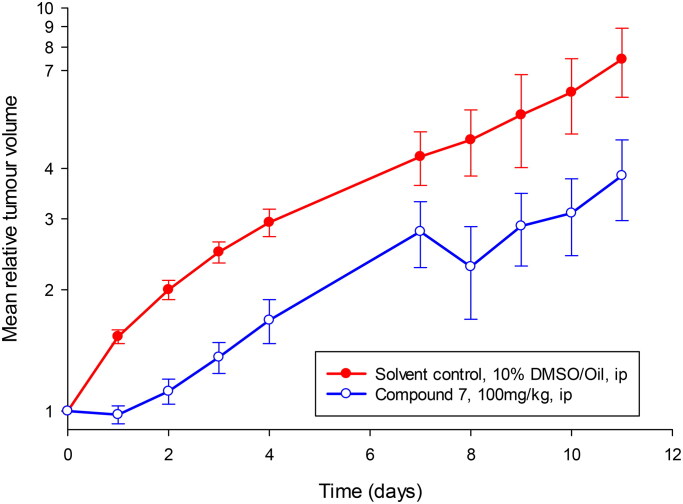
Antitumour activity of compound 7 against human A2780 ovarian tumour model *in vivo*. Mice bearing A2780 tumours were administered with an intraperitoneal dose of **7** (100 mg/kg) on days 0–4 and 7–11. The tumour growth delay, defined as time taken for doubling of tumour volume relative to control, was determined to be a significant delay of 3.7 days with **7**.

Given its promising *in vitro* activity and modelling profile, compound **7** was subsequently assessed *in vivo* for antitumour efficacy and systemic toxicity, as in previous studies with entinostat^90^. No systemic toxicity was detected for **7** when administered for 5 consecutive days as a single intraperitoneal dose, at a maximum tolerated dose of 100 mg/kg (data not shown). In terms of antitumour activity, administration of the maximum tolerated dose of 100 mg/kg for **7** (defined in this study) resulted in a significant delay in growth of the human ovarian A2780 tumour xenograft model of 3.7 days compared to the control groups (*p* < 0.01; Figure 9). No significant weight loss or overt toxicity was evident throughout the treatment period (Supplementary Figure S5). Together this strongly supports both *in vivo* pharmacological stability and good therapeutic efficacy of **7**, with evident potential for clinical translation of this series of KDACi.

## Conclusions

In conclusion, we describe development of a novel series of class I KDAC selective inhibitors, incorporating a vinyl group within the linker region and modifications of the capping group to exploit differences in the exit region of the KDAC active site. All compounds exhibited an ability to inhibit KDAC activity and potent antitumour activity against a large number of tumour types, with positional modifications of the trifluoromethyl group on the aromatic capping group demonstrating exquisite impacts upon their antitumour profile. For instance, location of this group at the ortho (**3**) and para (**5**) position commanded anti-leukaemic activity, whereas its presence at the meta position (**4**) afforded activity against a range of solid tumour types. Introduction of a pyridine capping group (**6**), as is present in the benchmark class I selective KDACI Entinostat (**1**), surprisingly resulted in diminished KDAC inhibitory ability and cytotoxic activity compared to other compounds. However, introduction of a trifluoromethyl group at the 2-position of the pyridine ring (**7**), analogous to that present in **4**, reinstated its activity and in fact resulted in improved activities. This compound presented selectivity towards HDAC1 over the other class I KDACs, HDAC2 and HDAC3, as is observed for Entinostat (**1**), but induced a significantly greater induction of histone acetylation than **1**. In terms of antitumour activity, compounds with the 3-trifluoromethyl aromatic (**4**) or the 2-trifluoromethyl substituted pyridine (**7**) capping group exhibited a much greater cytotoxicity profile than **1**, with the additional benefit of demonstrable activity against KDACi “resistant” and clinically aggressive representative tumour cell lines. Importantly, this activity translated *in vivo* with demonstrated antitumour efficacy of **7** against an ovarian tumour, with negligible evidence of toxicity, supporting the clinical potential of these novel chemotherapeutics. The differential activity of the most active compound in this series (**7**) relative to **1**, in terms of KDAC inhibitory ability and selectivity and cytotoxicity, can be explained by the differential orientation of these compounds within the active site of the KDAC enzyme. Modelling suggests they position differently along the hydrophobic channel of the active site and at the topological surface, with **1** aligning to the opposing surface of the active site exit to **7**. Such a difference may afford differential KDAC selectivities and affinities to these compounds, especially considering the clear variation in binding energies predicted for these compounds. Overall, these results indicate the importance of orientation of the KDACi within the active site and positioning of its capping group at the surface domain of the enzymatic active site for both KDAC enzyme selectivity and antitumour therapeutic activity and clinical potential. Together these data offer a new dimension which will lead the design efforts for new potent enzyme-selective KDAC inhibitors, with scope for management of cancers and other diseases.

## Supplementary Material

Gill_et_al_05_2025rev2_final_tracked- supplementary__.docx

## Data Availability

CCDC 2353836 contains the full crystallographic data for this article. These are available free of charge at www.ccdc.cam.ac.uk/data_request/cif, by emailing data_request@ccdc.cam.ac.uk or by contacting The Cambridge Crystallographic Data Centre, 12 Union Road, Cambridge, CB2 1EZ, UK; fax: +44 1223 336033
