# Supplementary material for: Development of novel benzamide class I selective lysine deacetylase inhibitors as potent anticancer agents
Source: J Enzyme Inhib Med Chem. 2025 Jul 1;40(1):2520612. doi: 10.1080/14756366.2025.2520612 (PMC12217109; doi:10.1080/14756366.2025.2520612)
Supplement: Gill_et_al_05_2025rev2_final_tracked- supplementary__.docx [file IENZ_A_2520612_SM6093.docx]

**Supplementary Material:**

**Development of Novel Benzamide Class I Selective Lysine Deacetylase Inhibitors as Potent Anticancer Agents**

Jason H Gill^1,2*^, Jonathan D. Sellars^1,3^, Paul G. Waddell^4^, Steven D Shnyder^5^, Ronald Grigg^6^, Colin W.G. Fishwick^6^

^1^School of Pharmacy, ^2^Translational and Clinical Research Institute, ^3^Biosciences Institute, Faculty of Medical Sciences, Newcastle University, UK.

^4^Chemistry, School of Natural and Environmental Science, Newcastle University, UK.

^5^Institute of Cancer Therapeutics, School of Life Sciences, University of Bradford, UK

^6^School of Chemistry, University of Leeds, UK

*Correspondence: E-mail: [Jason.gill@newcastle.ac.uk](mailto:Jason.gill@newcastle.ac.uk)

**CONTENTS:**

**Tables**

- **Supplementary Table 1:** Calculated physiochemical profiles of compounds 1-7
- **Supplementary Table 2:** ADME properties of compounds 1-7 predicted *in silico*
- **Supplementary Table 3:** Computational *de novo* design software module description
- **Supplementary Table 4:** NCI/NIH COMPARE data for compounds 1-7

**Figures**

- **Supplementary Figure 1:** NMR **s**pectra of compound 7
- **Supplementary Figure 2:** Graphical data in support of KDAC enzyme inhibition calculations
- **Supplementary Figure 3:** Flow cytometric graph supporting lack of tubulin acetylation by compound 7
- **Supplementary Figure 4:** Time dependent increase in Histone H4 acetylation induced by compound 7
- **Supplementary Figure 5:** Mouse body weight data associated with efficacy study.

**References**

|  |  |  |  |  |  |  |  | Drug-likeness | |
| --- | --- | --- | --- | --- | --- | --- | --- | --- | --- |
|  | **MW (Da)** | **nHBD** | **nHBA** | **LogP** | **N_Vio_** | **TPSA (A^2^)** | **nRotB** | **Lipinski^2^** | **Veber** |
| Compound | <500 | <5 | <10 | ≤5 | <1 | <140 | <10 | Yes/No | |
| (*Entinostat*) 1 | 376.42 | 4 | 7 | 2.03 | 0 | 106.34 | 7 | Yes | Yes |
| (*Tacedinaline*) 2 | 269.30 | 4 | 5 | 1.48 | 0 | 84.22 | 3 | Yes | Yes |
| 3 | 480.53 | 3 | 5 | 5.08 | 1 | 61.60 | 7 | No | Yes |
| 4 | 480.53 | 3 | 5 | 5.11 | 1 | 61.60 | 7 | No | Yes |
| 5 | 480.53 | 3 | 5 | 5.13 | 1 | 61.60 | 7 | No | Yes |
| 6 | 413.52 | 3 | 6 | 3.34 | 0 | 74.49 | 6 | Yes | Yes |
| 7 | 481.52 | 3 | 6 | 4.30 | 0 | 74.49 | 7 | Yes | Yes |

**Table S1: Physiochemical Profiles of Compounds Calculated using Molinspiration** (<https://www.molinspiration.com>)

MW, Molecular weight; nHBD, Number of H-bond donors; nHBA, Number of H-bond acceptors; LogP, Logarithm of partition coefficient of the compounds tested between n-octanol and water; Nvio, Number of Lipinski Rule of 5 (RO5) ^2^ violated; TPSA, Topological Polar Surface Area; nRotB, Number of rotatable bonds.

| Compound | Absorption | | | CYP Metabolism^b,c^ | | | | | | | Excretion^c^ | Toxicity^c^ |
| --- | --- | --- | --- | --- | --- | --- | --- | --- | --- | --- | --- | --- |
|  |  |  |  | **Substrate** | | **Inhibition** | | | | |  |  |
|  | **Log S^a,b^** *Water*  *(g/L)* | **Log S^b^**  *Buffer pH7.4*  *(g/L)* | **HIA^b^** | **2D6** | **3A4** | **2D6** | **3A4** | **1A2** | **2C19** | **2C9** | **Cl_tot_** | **hERG Inhibition** |
| (*Entinostat*) 1 | -3.81 (*0.06*) | -3.17 (*0.25*) | 92.5 | N | Y | N | Y | Y | Y | Y | 0.14 | No |
| (*Tacedinaline*) 2 | -2.84 (*0.40*) | -2.02 (*2.57*) | 89.9 | N | N | N | N | Y | N | N | 0.09 | No |
| 3 | -6.04 (*0.44x10^-3^*) | -6.59 (*0.12x10^-3^*) | 95.9 | Y | Y | Y | N | N | N | N | 0.61 | Yes |
| 4 | -6.09 (*0.39x10^-3^*) | -6.56 (*0.13x10^-3^*) | 95.9 | Y | Y | Y | N | N | N | N | 0.68 | Yes |
| 5 | -6.11 (*0.37x10^-3^*) | -6.34 (*0.22x10^-3^*) | 95.9 | Y | Y | Y | N | N | Y | N | 0.73 | Yes |
| 6 | -4.82 (*6.26x10^-3^*) | -5.85 (*0.58x10^-3^*) | 95.7 | Y | Y | Y | Y | N | Y | Y | 0.87 | No |
| 7 | -5.85 (*0.68x10^-3^*) | -6.04 (*0.44x10^-3^*) | 95.8 | Y | Y | Y | N | N | N | N | 0.56 | No |

**Table S2: ADME properties of compounds predicted *in silico***

Water solubility Log S (mol/L); Human intestinal absorption, HIA (%); Plasma Protein Binding, PPB (%); Substrate, S; Inhibitor, I; Total Clearance, Cl_tot_ (Log ml/min/kg).

^a^Calculated using SwissADME^3^;

^b^Calculated using preADMET^4^;

^c^Calculated with psKCM^5^

| **Module Name** | **Module Description** |
| --- | --- |
| **CANGAROO** | **C**left **AN**alysis by **G**eometry based **A**lgorithm **R**egardless **O**f the **O**rientation. In this module the receptor site and cavity (ligand) are defined. |
| **HIPPO** | **H**ydrogen-bonding **I**nteraction site **P**rediction as **P**ositions with **O**rientations.  In this module, potential binding sites are outlined, such as amino acids that can provide hydrogen bonding capability (either donating or accepting). The hydrophobic regions and metal interactions are also defined in this module. |
| **ELEFANT** | **ELE**ction of **F**unctional groups and **AN**choring them to **T**arget sites.  In this module, the fragments that contain functionality for hydrogen-bonding are identified and assigned to the complementary target sites. |
| **SPIDER** | **S**tructure **P**roduction with **I**nteractive **DE**sign of **R**esults.  In this module, spacer templates are selected to link the fragments which were selected in ELEFANT. Within the constraints of the target site and boundary surface, structures are then generated. |
| **ALLIGATOR** | **A**nalyse **L**ots of **LIGA**nds, **T**est and **O**rder **R**esults.  In this module, molecules are clustered into groups based on parameters set by the user, such as rotatable bonds, hydrophobic interactions and hydrogen-bonding interactions. |

**Table S3: Description of the modules within the *de novo* design software SPROUT, used in the design of novel drug-like molecules. ^1^**

**Table S4.1:** NCI/NIH COMPARE data for compound 1 (entinostat; NSC756642) against the NCI standard agent and synthetic database

|  | Drug target | Pearson’s correlation coefficient |
| --- | --- | --- |
| NSC 760143 (Mocetinostat)   | HDAC | 0.77 |
| NSC75435 (Buparlisib)   | PI3K | 0.70 |

**Table S4.2:** NCI/NIH COMPARE data for compound 3 (NSC740121) against the NCI standard agent and synthetic database

| Compound | Drug target | Pearson’s correlation coefficient |
| --- | --- | --- |
| NSC641818   |  | 0.84 |
| NSC709991   |  | 0.82 |
| NSC675989   |  | 0.82 |
| NSC683483   |  | 0.81 |
| NSC680072   |  | 0.80 |
| NSC674912   |  | 0.80 |
| NSC740122   |  | 0.80 |
| NSC671110   |  | 0.79 |
| NSC735408   | HDAC | 0.79 |
| NSC736101   | HDAC | 0.78 |
| NSC286161   |  | 0.78 |
| NSC708094   |  | 0.78 |
| NSC791140   |  | 0.78 |
| NSC169517   |  | 0.78 |
| NSC709748   |  | 0.78 |
| NSC29228   |  | 0.77 |
| NSC82365   | Notch | 0.77 |
| NSC703020   |  | 0.77 |
| NSC627740   |  | 0.77 |
| NSC742801   |  | 0.77 |
| NSC735182   |  | 0.77 |
| NSC689729   |  | 0.76 |
| NSC697134   |  | 0.76 |
| NSC380804   |  | 0.76 |
| NSC713797   |  | 0.76 |
| NSC657320   |  | 0.76 |
| NSC609694   |  | 0.75 |
| NSC684424   |  | 0.75 |
| NSC662824   |  | 0.75 |
| NSC707068   |  | 0.75 |
| NSC637436   |  | 0.75 |
| NSC689740   |  | 0.75 |
| NSC685301   |  | 0.74 |
| NSC685405   |  | 0.74 |
| NSC369318   |  | 0.74 |
| NSC676316   |  | 0.74 |
| NSC368253   |  | 0.74 |
| NSC784216   |  | 0.74 |
| NSC648147   |  | 0.74 |
| NSC735172   |  | 0.74 |
| NSC657026   |  | 0.74 |
| NSC71543   |  | 0.73 |
| NSC678503   |  | 0.73 |
| NSC68491   |  | 0.73 |
| NSC665680   |  | 0.73 |
| NSC30712   |  | 0.73 |
| NSC690559   |  | 0.73 |
| NSC685107   |  | 0.73 |
| NSC744999   |  | 0.73 |
| NSC677938   |  | 0.73 |
| NSC625156   |  | 0.73 |
| NSC88871   |  | 0.73 |
| NSC645159   |  | 0.73 |
| NSC208733   |  | 0.72 |
| NSC709882   |  | 0.72 |
| NSC707081   |  | 0.72 |
| NSC744647   |  | 0.72 |
| NSC775463   |  | 0.72 |
| NSC665348   |  | 0.72 |
| NSC707042   |  | 0.72 |
| NSC2186   |  | 0.72 |
| NSC670140   |  | 0.72 |
| NSC708426   |  | 0.72 |
| NSC740051   |  | 0.71 |
| NSC638352   |  | 0.71 |
| NSC681082   |  | 0.71 |
| NSC162062   |  | 0.71 |
| NSC668605   |  | 0.71 |
| NSC645656   |  | 0.71 |
| NSC73109   |  | 0.71 |
| NSC641393   |  | 0.71 |
| NSC685468   |  | 0.71 |
| NSC617806   |  | 0.71 |
| NSC234214   |  | 0.71 |
| NSC685105   |  | 0.71 |
| NSC717478   |  | 0.71 |
| NSC735404   | HDAC | 0.71 |
| NSC96914   |  | 0.71 |
| NSC704402   |  | 0.71 |
| NSC73754   | Alkylating agent | 0.71 |
| NSC382000   |  | 0.70 |
| NSC709753   |  | 0.70 |
| NSC740108   |  | 0.70 |
| NSC99300   |  | 0.70 |
| NSC715448   |  | 0.70 |
| NSC624625   |  | 0.70 |
| NSC681152   |  | 0.70 |
| NSC669503   |  | 0.70 |
| NSC675210   |  | 0.70 |
| NSC678035   |  | 0.70 |
| NSC662383   |  | 0.70 |
| NSC342731   |  | 0.70 |
| NSC684397   |  | 0.70 |
| NSC665350   |  | 0.70 |

**Table S4.3:** NCI/NIH COMPARE data for compound 4 (NSC730003) against the NCI standard agent and synthetic database

| Compound | Drug target | Pearson’s correlation coefficient |
| --- | --- | --- |
| NSC 730594   |  | 0.70 |

**Table S4.4:** NCI/NIH COMPARE data for compound 5 (NSC740122) against the NCI standard agent and synthetic database

| Compound | Drug target | Pearson’s correlation coefficient |
| --- | --- | --- |
| NSC740121 |  | 0.80 |
| NSC685301 |  | 0.77 |
| NSC59267 |  | 0.75 |
| NSC637436 |  | 0.75 |
| NSC165839 |  | 0.73 |
| NSC82365 | Notch | 0.73 |
| NSC169517 |  | 0.73 |
| NSC736101 | HDAC | 0.72 |
| NSC684913 |  | 0.72 |
| NSC735408 | HDAC | 0.72 |
| NSC661110 |  | 0.72 |
| NSC735004 |  | 0.72 |
| NSC641818 |  | 0.71 |
| NSC101335 |  | 0.71 |
| NSC668606 |  | 0.71 |
| NSC684424 |  | 0.71 |
| NSC684876 |  | 0.71 |
| NSC686140 |  | 0.71 |
| NSC150316 |  | 0.71 |
| NSC703020 |  | 0.71 |
| NSC71297 |  | 0.71 |
| NSC685107 |  | 0.70 |
| NSC684850 |  | 0.70 |
| NSC711759 |  | 0.70 |
| NSC26821 |  | 0.70 |
| NSC34931 |  | 0.70 |

**Table S4.5:** NCI/NIH COMPARE data for compound 6 (NSC729999) against the NCI standard agent and synthetic database

| Compound | Drug target | Pearson’s correlation coefficient |
| --- | --- | --- |
| - | - | - |

**Table S4.6:** NCI/NIH COMPARE data for compound 7 (NSC742611) against the NCI standard agent and synthetic database

| Compound | Drug target | Pearson’s correlation coefficient |
| --- | --- | --- |
| NSC812228 |  | 0.76 |
| NSC810517 |  | 0.76 |
| NSC810513 |  | 0.75 |
| NSC745452 |  | 0.75 |
| NSC796238 |  | 0.74 |
| NSC755424 |  | 0.74 |
| NSC809940 |  | 0.73 |
| NSC804262 |  | 0.73 |
| NSC809872 |  | 0.73 |
| NSC759882 |  | 0.72 |
| NSC809856 |  | 0.72 |
| NSC809548 |  | 0.72 |
| NSC801519 |  | 0.72 |
| NSC810520 |  | 0.72 |
| NSC801548 |  | 0.72 |
| NSC800347 |  | 0.72 |
| NSC801886 |  | 0.72 |
| NSC805641 |  | 0.72 |
| NSC810447 |  | 0.71 |
| NSC281383 |  | 0.71 |
| NSC795903 |  | 0.71 |
| NSC750106 |  | 0.71 |
| NSC747339 |  | 0.71 |
| NSC30813 |  | 0.70 |
| NSC803281 |  | 0.70 |
| NSC809747 |  | 0.70 |

**Figure S1:** ^1^H NMR spectrum of compound 7

**Figure S2: HDAC inhibition profiles of compounds, determined by Fluor-de-Lys assay**

**
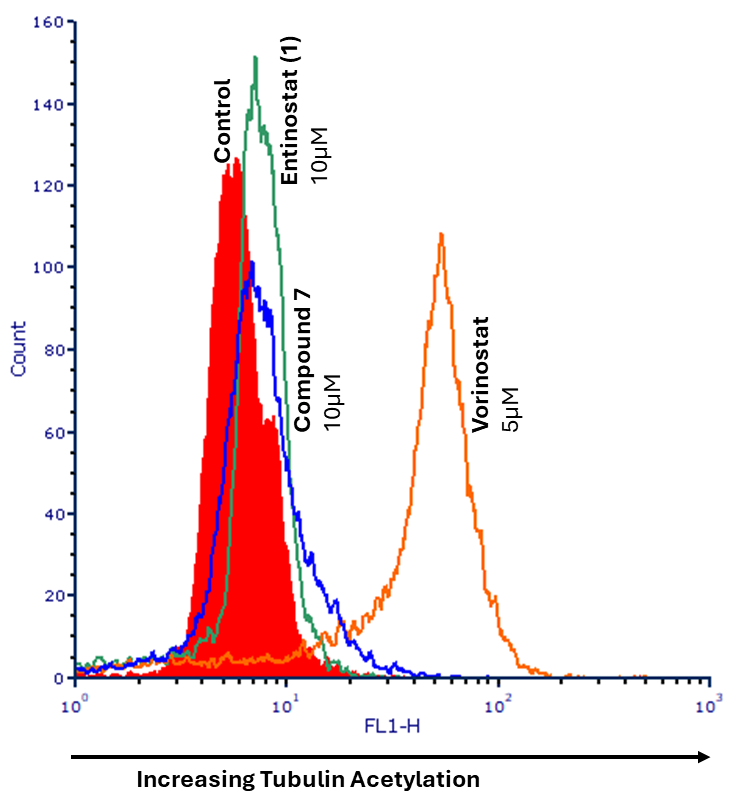
**

**Figure S3: No change in Tubulin acetylation status by compound 7 or entinostat (1) compared to reference compound Vorinostat.** Flow cytometric histogram depicting relative increase of acetylated tubulin following a 24-hour exposure of the A2780 ovarian cell line to either compound **7** (10µM; Blue line), Entinostat (10µM; Green line) or the hydroximate KDACi vorinostat/suberoylanilide hydroxamic acid (SAHA) (5µM; Orange line).


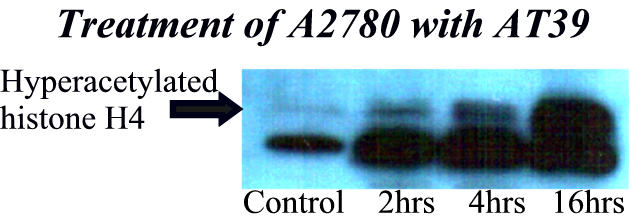


**Figure S4: Time dependent increase in Histone H4 acetylation induced by compound 7.** The human A2780 ovarian cancer cell was incubated *in vitro* with 10 µM of **7** and the cells lysed at specific timepoints (2, 4, 16 hrs). The levels of histone acetylation were thereafter detected by immunoblotting.

**Figure S5: Administration of compound 7 did not induce significant weight loss or detrimental toxicity upon mice bearing human A2780 ovarian tumour models *in vivo.***

Mice bearing A2780 tumours were administered with an intraperitoneal dose of **7** (100 mg/kg) on days 0-4 and 7-11. The bodyweight of mice on the study were compared to those at the point of compound administration, across the duration of the study. No significant reduction in bodyweight was observed in mice treated with compound 7, relative to those administrated drug vehicle (DMSO/Oil) alone.

**References**

1. Simmons KJ, Chopra I, Fishwick CW. Structure-based discovery of antibacterial drugs. Nat Rev Microbiol 2010:8(7): 501-510.

2. Lipinski CA, Lombardo F, Dominy BW, Feeney PJ. Experimental and computational approaches to estimate solubility and permeability in drug discovery and development settings. Adv Drug Deliv Rev 2001:46(1-3): 3-26.

3. Daina A, Michielin O, Zoete V. Swissadme: A free web tool to evaluate pharmacokinetics, drug-likeness and medicinal chemistry friendliness of small molecules. Sci Rep 2017:7:42717.

4. Lee S, Chang G, Lee I, Chung J, Sung K, No K. The preadme: Pc-based program for batch prediction of adme properties. EuroQSAR 2004:9:5-10.

5. Pires DE, Blundell TL, Ascher DB. Pkcsm: Predicting small-molecule pharmacokinetic and toxicity properties using graph-based signatures. J Med Chem 2015:58(9): 4066-4072.
